# Vulnerability of fault-tolerant topological quantum error correction to quantum deviations in code space

**DOI:** 10.1093/pnasnexus/pgaf063

**Published:** 2025-02-25

**Authors:** Yuanchen Zhao, Dong E Liu

**Affiliations:** State Key Laboratory of Low Dimensional Quantum Physics, Department of Physics, Tsinghua University, Beijing 100084, People’s Republic of China; Frontier Science Center for Quantum Information, Beijing 100184, People’s Republic of China; State Key Laboratory of Low Dimensional Quantum Physics, Department of Physics, Tsinghua University, Beijing 100084, People’s Republic of China; Frontier Science Center for Quantum Information, Beijing 100184, People’s Republic of China; Beijing Academy of Quantum Information Sciences, Beijing 100193, People’s Republic of China; Hefei National Laboratory, Hefei 230088, People’s Republic of China

## Abstract

Quantum computers face significant challenges from quantum deviations or coherent noise, particularly during gate operations, which pose a complex threat to the efficacy of quantum error correction (QEC) protocols. Here we scrutinize the performance of the topological toric code in 2D under the dual influence of stochastic noise and quantum deviations, especially during the critical phases of initial state preparation and error detection facilitated by multiqubit entanglement gates. By mapping the multiround error detection protocol—from the inception of an imperfectly prepared code state via imperfect stabilizer measurements—to a statistical mechanical model (3D $Z_{2}$ gauge theory coupled with 2D $Z_{2}$ gauge theory), we establish a link between the error threshold and the model’s phase transition. Specifically, we find two distinct error thresholds that demarcate varying efficacies in QEC. The empirical threshold that signifies the operational success of QEC aligns with the theoretical ideal of flawless state preparation operations. Contrarily, below another finite theoretical threshold, a phenomenon absent in purely stochastic error models emerges: unidentifiable measurement errors precipitate QEC failure in scenarios with large code distances. For codes of finite distance *d*, it is revealed that maintaining the preparation error rate beneath a crossover scale, proportional to 1/logd, allows for the suppression of logical errors. Considering that fault-tolerant quantum computation is valuable only in systems with large scale and exceptionally low logical error rates, this investigation explicitly demonstrates the serious vulnerability of fault tolerant QEC based on 2D toric codes to quantum deviations in code space, highlighting the imperative to address inherent preparation noise.

**Significance Statement**

Our findings address the challenge of mitigating both classical and quantum noise in quantum computers. By bridging quantum error correction (QEC) protocols with novel lattice gauge models, we offer insights into the intricate interplay of noise types and their impact on fault-tolerant quantum computation. Surprisingly, we unveil that the presence of quantum (coherent) noise during state preparation undermines QEC efficacy even below a newly identified theoretical threshold, challenging established beliefs about error threshold theorem. This investigation explicitly demonstrates the serious vulnerability of fault tolerant QEC to quantum deviations in code space, highlighting the imperative to address inherent preparation noise.

## Introduction

Quantum supremacy was recently observed in quantum processors (1–3), which is a milestone in the field of quantum computation. However, the state-of-the-art quantum devices (1–8) are classified as noisy intermediate-scale quantum (NISQ) (9) computer, and the observed quantum supremacy is only a weakened version with few practical applications (9). To date, the merely known examples with worthwhile quantum advantages are only expected in fault tolerant quantum computers with quantum error correction (QEC) (10–12). Recently, QEC codes with small size have recently been tested in experiments (6, 8, 13–24).

A cornerstone of fault tolerance is the “error threshold theorem.” It posits that if the physical error rates across all facets of quantum computation—including code state preparation, stabilizer checks, logical operations, and readout—remain beneath a finite threshold, then one can achieve arbitrary logical accuracy within a noisy quantum device (25–27). The threshold theorem is well established if the device noise can be captured by independent stochastic errors (25, 27–33), including circuit-level noise models (34, 35). However, actual quantum devices suffer from more general type of errors. With correlated errors, the threshold theorem is modified for a more conceptual infidelity measure, e.g. diamond norm (26), for correlations with weak amplitude (36) and short length (37), and for the environment with critical behaviors (38, 39). A more practical type of noise comes from imperfect calibration and control of gate operations, causing quantum deviation or coherent effect in errors. This problem motivated recent studies of the independent single-qubit coherent errors (40–48) and the detection induced coherent errors from entanglement gate noise (49–51). We emphasize that the two-qubit entanglement gates are much harder to calibrate and more error-prone than single-qubit gates. Comprehending the fault-tolerance and error threshold theorem in practical quantum systems exhibiting coherent deviations proves to be formidable, primarily due to the scarcity of analytical and numerical methodologies. Although an efficient numerical strategy exists for a special case (42), the general coherent error problems, that go beyond the Clifford algebra, cannot be simulated efficiently in classical computers. Additionally, previous studies on QEC error thresholds have seldom addressed quantum deviations in code state preparation and error detection. This motivates us to develop a theoretical framework to study the elusive and unavoidable quantum deviations alone with stochastic errors in realistic QEC procedures and establish a more practical error threshold theorem.

### Summary of main results

**Fig. 1.**
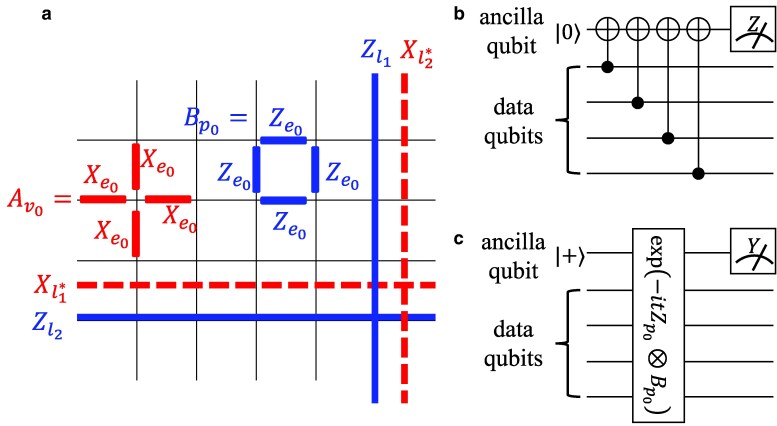
a) Toric code defined on 2D periodic lattice. Physical qubits stay on the edges of the lattice. The two kinds of stabilizers are $A_{v_{0}}=\prod_{e_{0}|v_{0}\in \partial e_{0}}X_{e_{0}}$ defined on each vertex and $B_{\;p_{0}}=\prod_{e_{0}\in \partial p_{0}}Z_{e_{0}}$ defined on each plaquette as shown in the figure. $l_{1}$ and $l_{2}$ denote noncontractible loops on the periodic lattice. The logical Pauli *Z* operators $Z_{l_{1}}$ and $Z_{l_{2}}$ are product of *Z*’s along these noncontractible loops. Correspondingly the logical Pauli *X* operators $X_{l_{1}^{*}}$ and $X_{l_{2}^{*}}$ are defined as *X*’s along noncontractible loops on the dual lattice $l_{1}^{*}$ and $l_{2}^{*}$. They satisfy the commutation relations $X_{l_{1}^{*}}Z_{l_{1}}=-Z_{l_{1}}X_{l_{1}^{*}}$, $X_{l_{1}^{*}}Z_{l_{1}}=-Z_{l_{1}}X_{l_{1}^{*}}$, $X_{l_{1}^{*}}Z_{l_{2}}=Z_{l_{2}}X_{l_{1}^{*}}$, $X_{l_{2}^{*}}Z_{l_{1}}=Z_{l_{1}}X_{l_{2}^{*}}$ and acts, respectively on two different logical qubits of toric code. b) A realistic circuit for $B_{\;p_{0}}$ measurement (29). The ancilla qubit is prepared in |0⟩ state, then four *CNOT* gates are applied in order to couple data and ancilla qubits. Finally, the ancilla is projectively measured in *Z* basis. c) A simplified $B_{\;p_{0}}$ measurement circuit considered in our work. Note that a five-qubit unitary gate is used here instead of four two-qubit gates. To enable a theoretical analysis of the problem, we focus on the case (c) in our model: (1) Prepare the ancilla qubit in |+⟩ state for each plaquette $p_{0}$; (2) apply a joint time evolution involving each ancilla and its four neighboring data qubits $\exp\;[-itZ_{\;p_{0}}⊗B_{\;p_{0}}]$ where $Z_{\;p_{0}}$ is the Pauli *Z* acting on ancilla at $\;p_{0}$; (3) perform projective measurement on ancilla in *Y* basis. This model is a rather simplified one which is easier to study analytically, but it can capture the fundamental influence of imperfect stabilizer measurement on QEC. A more realistic imperfect measurement model relating to (b) will in general has a worse performance, see the discussion of Eq. 24. A similar construction can also be applied to $A_{v}$ stabilizers.

In this work, we study the robustness of the toric code QEC (52), under the influence of imperfect state preparation and measurements, as visualized in Fig. 1, along with stochastic Pauli errors afflicting physical qubits. We consider the realistic case that the measurement apparatus, crucial for both the preparation of the initial state and the subsequent error detection steps, is compromised by coherent noise. The initialization of code states is facilitated through an initial round of stabilizer measurements, following which these states are subjected to an error correction regimen, where the common multiround syndrome measurements method (28, 29) is used to decode errors. Such a procedure is also adopted by experiments (23).

Further, we transition the operational framework of this QEC protocol into a novel statistical mechanical (SM) model, characterized by the integration of a 3D $Z_{2}$ gauge theory with a 2D $Z_{2}$ gauge theory. This integration is achieved via a distinctive, nonlocal timelike coupling originates from the imperfection during initial state preparation. This conceptual model serves as the basis for our exploration into the interaction dynamics between QEC processes and the pervasive influences of coherent noise and stochastic errors, thereby offering novel insights into the resilience and limitations of the toric code QEC methodology under practical conditions. We also emphasize the theoretical novelty in deriving an explicit SM model with coherent errors, as typically, such models are limited to stochastic Pauli errors and Clifford structures.

We find that the Wilson loops in this SM model have an anisotropic behavior: The timelike Wilson loops deconfine at low temperatures (small physical error rates) and confine at high temperatures (large physical error rates); but the spacelike Wilson loops confine at any finite temperature, resulting from the nonlocal timelike correlation. Then, we predict that there are two thresholds in our QEC model. The confinement–deconfinement transition point of timelike Wilson loops signifies a theoretical threshold located at finite measurement error rate and finite Pauli error rate. However, the confinement behavior of spacelike Wilson loops suggests that the practical measurement error threshold seats at the point where the initial state preparation is perfect. With arbitrary finite preparation error, the measurement errors will no longer be distinguished from Pauli errors, which results in the failure of QEC even in the limit of large code distance. With a finite code distance *d*, the effectiveness of the pragmatic QEC approach remains viable when the error rate associated with state preparation falls within a region ∼1/logd. Finally, we emphasize that a more realistic imperfect measurement model relating to Fig. 1b will in general has a worse performance.

## Model for toric code under imperfect measurement

We follow the construction of topological surface code on a torus, i.e. toric code (28, 29, 32, 52). There are two kinds of stabilizers associated with vertices and plaquettes, respectively as shown in Fig. 1a,


(1) $$A_{v_{0}}=\prod_{e_{0}|v_{0}\in \partial e_{0}}X_{e_{0}},\;B_{\;p_{0}}=\prod_{e_{0}\in \partial p_{0}}Z_{e_{0}}.$$

Here we use the symbols $v_{0}$ and $p_{0}$ to label vertex and plaquette operators. $X_{e_{0}}$ and $Z_{e_{0}}$ represent Pauli operators acting on qubit $e_{0}$ (i.e. “edge”). We assume the lattice contains *N* vertices, *N* plaquettes and 2N edges. 2N physical qubits are put on each edge of the lattice. Its 4D code subspace is stabilized by all $A_{v_{0}}$’s and $B_{\;p_{0}}$’s which is achieved through projective measurement of these stabilizers. Specifically, we start with the logical ++ state by projecting all the $B_{\;p_{0}}$’s to +1 for a product state of physical qubits $\underset{e_{0}}{⨂}|+\rangle_{e_{0}}$:


(2) $$|++\rangle =\prod_{\;p_{0}}\frac{I+B_{\;p_{0}}}{2}\underset{e_{0}}{⨂}|+\rangle_{e_{0}}$$

and other three logical bases are obtained by applying logical Pauli *Z* operators $\{Z_{l_{1}}$, $Z_{l_{2}}\}$ as shown in Fig. 1a, and these four logical bases form a code subspace C. Correspondingly there are also logical Pauli *X* operators $\left\{X_{l_{1}^{*}},X_{l_{2}^{*}}\right\}$.

Experimentally, the stabilizer measurements are implemented using a multiqubit unitary operation on a combined qubit set consisting of four data qubits and an ancilla qubit, followed by an ancilla qubit measurement (29, 53), also refer to Fig. 1b. The correct projective measurements of stabilizers can only be achieved through ideal unitary operations. However, the multiqubit operation in principle cannot avoid the miscalibration in the experimental setups and results in imperfect measurement (50). Here we consider a simplified imperfect measurement model (51) with a joint time evolution involving each ancilla and its four neighboring data qubits $\exp\;[-itZ_{\;p_{0}}⊗B_{\;p_{0}}]$ (refer to Fig. 1c). Then equivalently we get an operator acting on the data qubits


(3) $$M_{\left\{s_{\;p_{0}}\right\}}=\frac{1}{(\sqrt{2\cosh\;\beta }{)}^{N}}\exp\;\left[\frac{1}{2}\beta \sum_{\;p_{0}}s_{\;p_{0}}B_{\;p_{0}}\right].$$

up to an irrelevant global phase factor. Note that $M_{\left\{s_{\;p_{0}}\right\}}$ is not a unitary operator. Here tanh(β/2)=tant, and $s_{\;p_{0}}=\pm 1$ is the measurement outcome of ancilla qubit at $p_{0}$. We use $\left\{s_{\;p_{0}}\right\}$ to denote the configuration of ancilla measurement outcomes, which appears with probability $\text{tr}(M_{\left\{s_{\;p_{0}}\right\}}\rho M_{\left\{s_{\;p_{0}}\right\}}^{†})$ for a given initial state *ρ* of data qubits. It is easy to verify that the $E_{\left\{s_{\;p_{0}}\right\}}=M_{\left\{s_{\;p_{0}}\right\}}^{†}M_{\left\{s_{\;p_{0}}\right\}}$ operators form a set of positive operator-valued measurement (POVM). The error model (51) only considers the miscalibration of the evolution time *t* with 0≤t≤π/4. When t=π/4, we have β→+∞ in Eq. 3 and recover the correct projective measurement $M_{\left\{s_{\;p_{0}}\right\}}\propto \prod_{\;p_{0}}(I+s_{\;p_{0}}B_{\;p_{0}})/2$. For t<π/4 the parameter *β* is finite, and $M_{\left\{s_{\;p_{0}}\right\}}$ will no longer be a stabilizer projection. Here we will assume the coherent deviation δt=t−π/4>0 to be a fixed value, while in Sec.SVI of supplemental information (SI) (54), we briefly discuss the case when it is randomly distributed. Generally, *β* measures how close is our imperfect measurement to the ideal projective measurement.

Experimentally while preparing the initial logical state through stabilizer measurement as in Eq. 2, the coherent noises are unavoidable, especially for the entanglement gates. For the imperfect measurement model we adopt (Eq. 3), the imperfect initial logical ++ state is considered as


(4) $$|\tilde{++}\rangle =\frac{M_{\left\{+\right\}}\underset{e_{0}}{⨂}|+\rangle_{e_{0}}}{\sqrt{\underset{e_{0}}{⨂}\langle +{|}_{e_{0}}M_{\left\{+\right\}}^{†}M_{\left\{+\right\}}\underset{e_{0}}{⨂}|+\rangle_{e_{0}}}},$$

where all the ancilla measurement outcomes $s_{\;p_{0}}$ are set to +1. We define the other three logical states by applying logical *Z* operators in analogy to experimental setups, $|\tilde{-+}\rangle =Z_{l_{1}}||\tilde{++}\rangle \rangle$, $|\tilde{+-}\rangle =Z_{l_{2}}|\tilde{++}\rangle$ and $|\tilde{--}\rangle =Z_{l_{1}}Z_{l_{2}}|\tilde{++}\rangle$. Note that those states are still orthogonal to each other, and define the corresponding code space $\tilde{C}(\beta )$ as the subspace spanned by the above four states (refer to Sec.SI of SI (54) for more details). Unlike the perfect measurement case, those logical states depend on the choice of logical operators about where they are located on the physical lattice. However, the key results presented in this work—the SM model and its implications to QEC—remain unaffected by the particular selection of logical operators.

These states are argued to be nontopological (51, 55) by a mapping to the 2D $Z_{2}$ lattice gauge theory. Such states may challenge QEC, yet the behavior of practical QEC procedures and the corresponding error thresholds remains an open question. Indeed, Sang et al. (56) suggest that in the presence of stochastic noise, the mixed-state topological phase transition happens before the recoverability transition, given that topological properties are defined by local operations and recovery can involve nonlocal operations. Additionally, for general imperfect initial codes considered as approximate QEC, Yi et al. (57) identify nontopological states that correct certain local errors in large systems. Consequently, it is essential to assess a specific QEC protocol with imperfect measurements and directly analyze its error threshold characteristics.

## SM mapping

Normally for toric code, the Pauli errors are detected by syndrome measurement. However, the syndrome measurement also suffers from imperfection resulting in faulty outcomes. Therefore, in order to distinguish measurement errors from Pauli errors, the standard procedure is to perform multirounds of syndrome measurements and take into account the obtained entire error history while decoding (28). Note that Dennis et al. (28) only consider the stochastic errors of the ancilla measurement and assume a well-prepared initial state from the perfect code space, but we consider imperfect entanglement operations which affect both the initial state preparation and the error detection. We model the QEC procedure as follows (for convenience, we consider only the sector from Pauli *X* errors):

1. Start with an arbitrary state $|\tilde{\Psi }\rangle \in \tilde{C}(\beta_{0})$, the imperfect measurement strength while preparing the initial state is $\beta_{0}$.
2. Probabilistic Pauli *X* error acts at each integer valued time t=−∞,…,−1,0,1,…,+∞. The *X* error at each physical qubit on each time slice occurs independently with probability q∈[0,1/2].
3. Perform a round of syndrome measurement for each time interval between *t* and t+1. The syndrome measurements are assumed to still suffer from imperfect measurement. So given a configuration of syndrome measurement outcomes $\left\{s_{\;p_{0}}\right\}$ for a single round, it leads to the action of $M_{\left\{s_{\;p_{0}}\right\}}$ operator on the current quantum state. Here we set the strength of syndrome measurements to be *β* in order to distinguish them from initial state preparation.
4. At the end of the QEC procedure, we decode and apply the Pauli *X* correction operator to the final state.

We first notice that our model ensures that the imperfection of measurement exclusively affects stabilizer bits while leaving logical information undisturbed, since $M_{\left\{s_{\;p_{0}}\right\}}$ commutes with logical operators. So, if the Pauli *X* errors and the final correction operator compose a contractible loop, we can verify that the logical information will still be preserved. We refer to this case as the success of QEC, in contrast to the situation with a noncontractible loop causing a logical error. Note that this condition for successful QEC is the same as the one in Ref. (28).

**Fig. 2.**
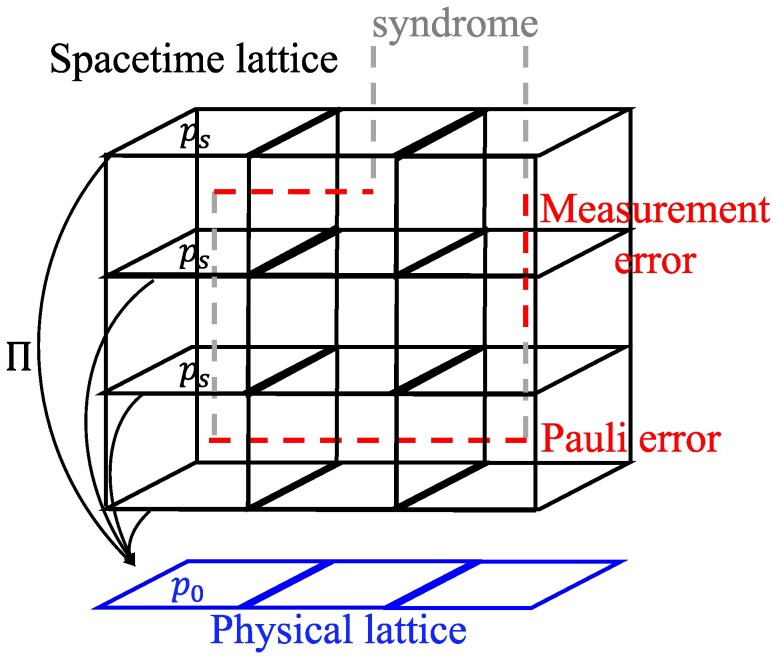
3D spacetime of error history. The black lattice is the spacetime lattice. At the spatial direction, we takes periodic boundary condition; at the temporal direction, we assume that the QEC starts at t=−∞ and ends at t=+∞, such that the 3D corresponding spacetime lattice has infinite boundary condition at time direction. These boundary conditions are also adopted in Ref. (28) when discussing SM mapping. The Pauli errors, measurement errors and error syndromes are represented as strings on the dual lattice (dashed lines that cross plaquettes). We use timelike and spacelike dual strings to represent measurement errors, i.e. faulty syndromes caused by imperfection of sequential measurements, and Pauli errors, respectively. Given a configuration of Pauli *X* error (horizontal highlighted strings) for the entire history, the error syndrome (vertical dimmed strings) will be the configuration of −1 ancilla measurement outcomes at different time steps. The error syndrome is supposed to match the endpoints of Pauli error strings, but due to imperfection of syndrome measurements, the syndrome outcomes might be flipped with certain probability. Those flipped syndromes will be referred to as measurement errors (vertical highlighted strings). *Π* denotes the projection from the 3D spacetime lattice to the 2D physical lattice. Given a plaquette $p_{s}$, $\Pi (p_{s})$ yields a plaquette $p_{0}$ at the same spatial location as $p_{s}$.

The above QEC procedure could be diagrammatically represented on a 3D cubic lattice in order to decode as in Fig. 2. The error strings (including both measurement and Pauli parts) and syndrome strings (marked with −1 ancilla outcomes) together compose closed strings or with only endpoints at infinity. The task of the decoder is to identify both measurement and Pauli errors. In order to do so, the decoder should select a configuration of strings (decoding strings) connecting the endpoints of syndrome strings. The timelike (spacelike) parts of the decoding strings represent the measurement (Pauli) error identified by the decoder. QEC succeeds if and only if the decoding strings are topologically equivalent to the real error strings (form contractible loops). So given an error syndrome, the optimal decoder algorithm (37) should select the topological equivalent class of error strings with the largest probability.

Our main result is that we mapped this QEC scenario to an statistical mechanical (SM) model. We denote the vertex, edge and plaquette of the 3D spacetime lattice as *v*, *e* and *p*. Specifically, the spacelike (timelike) edges and plaquettes will be labeled with a subscript *s* (*t*), such as $e_{s}$ and $p_{s}$ ($e_{t}$ and $p_{t}$). We assign a variable $\eta_{p}=\pm 1$ to each plaquette *p* to represent the error configuration, e.g. $\eta_{p}=-1$ for where the measurement or Pauli error presents and +1 otherwise. Then the probability of a given error configuration $\left\{\eta_{p}\right\}$ will be


(5) $$P(\{\eta_{p}\})=\frac{\sum_{\left\{\sigma_{e_{0}}\right\}}\exp\;\left[\beta_{0}\sum_{\;p_{0}}b_{\;p_{0}}+\beta \sum_{\;p_{s}}b_{\Pi (p_{s})}\eta_{\;p_{s}}+K\sum_{\;p_{t}}\eta_{\;p_{t}}\right]}{4^{N}(\cosh^{N}\;\beta_{0}+\sinh^{N}\;\beta_{0})(2\cosh\;\beta {)}^{NT}(2\cosh\;K{)}^{2NT}},$$

where


(6) $$\;b_{\;p_{0}}=\prod_{e_{0}\in \partial p_{0}}\sigma_{e_{0}},\;K=-\frac{1}{2}\log\;\frac{q}{1-q}.$$

Here $\sigma_{e_{0}}$ is a classical $Z_{2}$ spin-like variable assigned to each edge $e_{0}$ of the 2D physical lattice. $b_{\;p_{0}}$ is the product of four neighbouring $\sigma_{e_{0}}$’s around the plaquette $p_{0}$, which has the similar form to a $Z_{2}$ gauge interaction term (58, 59). $\Pi (p_{s})$ is the projection of the spacelike plaquette $p_{s}$ onto the 2D physical lattice, as in Fig. 2. The label *T* represents the total number of time steps, and will eventually be taken to +∞. The summation $\sum_{\left\{\sigma_{e_{0}}\right\}}$ runs over all $\left\{\sigma_{e_{0}}\right\}$ configurations.

The detailed derivation of Eq. 5 can be found in Sec.SII of SI (54), and we only list some key steps here. Notice that given a quantum state *ρ*, the probability of POVM outcome is $\text{tr}(E_{\left\{s_{\;p_{0}}\right\}}\rho )$. We may construct a probability of syndrome measurement outcomes of all time steps and space locations conditioned on a fixed Pauli error configuration, which is expressed as


(7) $$P\left(\{s_{\;p_{0}}(t){\}}_{t}|\{\eta_{e_{0}}(t){\}}_{t}\right)=\|\prod_{t}M_{\left\{s_{\;p_{0}}(t)\right\}}X_{\left\{\eta_{e_{0}}(t)\right\}}|\tilde{\Psi }\rangle {\|}^{2}$$

for an arbitrary initial state $|\tilde{\Psi }\rangle$ in the imperfect code space $\tilde{C}(\beta_{0})$. Specifically, any initial state should be a superposition of imperfect logical states $|\tilde{\Psi }\rangle =\Psi_{++}|\tilde{++}\rangle +\Psi_{+-}|\tilde{+-}\rangle +\Psi_{-+}|\tilde{-+}\rangle +\Psi_{--}|\tilde{--}\rangle$ and we assume it to be normalized. Here we introduced the label *t* to specify the time step. $\left\{s_{\;p_{0}}(t)\right\}$ and $\left\{\eta_{e_{0}}(t)\right\}$ denote the syndrome configuration and Pauli error configuration at time *t*, while $\{s_{\;p_{0}}(t){\}}_{t}$ and $\{\eta_{e_{0}}(t){\}}_{t}$ denote the configurations of the whole history. Note that a pair $\left(p_{0},t\right)$ yields a corresponding spacelike plaquette $p_{s}$ and a pair $\left(e_{0},t\right)$ yields a corresponding timelike plaquette $p_{t}$. $X_{\left\{\eta_{e_{0}}(t)\right\}}$ is the total Pauli error operator at time *t*, and has the form


(8) $$X_{\left\{\eta_{e_{0}}(t)\right\}}=\prod_{e_{0}}(\delta_{\eta_{e_{0}}(t),+1}I+\delta_{\eta_{e_{0}}(t),-1}X_{e_{0}}).$$

After combining with Pauli error probability


(9) $$\begin{array}{rl} P(\{\eta_{e_{0}}(t){\}}_{t}) & =\prod_{e_{0},t}q^{\delta_{\eta_{e_{0}}(t),-1}}(1-q{)}^{\delta_{\eta_{e_{0}}(t),+1}}\\ & =\prod_{e_{0},t}\frac{\exp\;(K\eta_{e_{0}}(t))}{2\cosh\;K}, \end{array}$$

we can obtain the joint probability of total error configurations $P(\{\eta_{\;p_{0}}(t){\}}_{t},\{\eta_{e_{0}}(t){\}}_{t})$, which is exactly Eq. 5 after converting the notations to those of the 3D lattice (we now denote $\{\eta_{p}\}=\{\eta_{\;p_{0}}(t),\eta_{e_{0}}(t){\}}_{t}$).

Then following the standard procedure described in Ref. (28, 32, 37), we computed the probability of the topological equivalent class of error configurations. by introducing the gauge interaction term $U_{p}$, the summation over topologically equivalent error configurations is converted to the summation of spin configurations $\left\{\tau_{e}\right\}$ up to a constant factor. The result is proportional to the partition function of an SM model


(10) $$\begin{array}{rl} & P([\{\eta_{\;p}\}])\propto Z(\{\eta_{p}\})\\ & \;=\sum_{\left\{\sigma_{e_{0}}\},\{\tau_{e}\right\}}\exp\;\left[\beta_{0}\sum_{\;p_{0}}b_{\;p_{0}}+\beta \sum_{\;p_{s}}b_{\Pi (p_{s})}\eta_{\;p_{s}}U_{\;p_{s}}+K\sum_{\;p_{t}}\eta_{\;p_{t}}U_{\;p_{t}}\right],\\ & U_{\;p}=\prod_{e\in \partial p}\tau_{e}. \end{array}$$

**Fig. 3.**
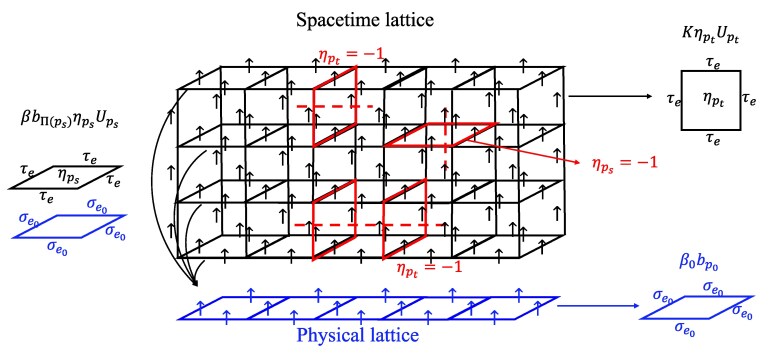
Illustration of the SM model we obtained in Eq. 10. The $\tau_{e}$ spins are defined on the edges of 3D spacetime lattice and the $\sigma_{e_{0}}$ spins lie on 2D physical lattice. There are three types of interactions in this model as shown in this figure. $\beta_{0}b_{\;p_{0}}$ is the gauge interaction term defined on the physical lattice. $K\eta_{\;p_{t}}U_{\;p_{t}}$ is the timelike gauge interaction on spacetime lattice. The $\beta b_{\Pi (p_{s})}\eta_{\;p_{s}}U_{\;p_{s}}$ term couples the spacelike gauge interaction term $U_{\;p_{s}}$ to the gauge interaction term $b_{\Pi (p_{s})}$ on physical lattice. The $\eta_{p}$’s set the signs of gauge interactions on spacetime lattice and they mark the position of error strings during the QEC procedure in Fig. 2. For example, the flipped interaction $\eta_{p}=-1$ at plaquette *p* (highlighted plaquette in 3D lattice) corresponds to the presence of an error string at *p* (dashed line). The $\left\{\eta_{p}\right\}$ configuration follows a disorder probability Eq. 5 that comes from the randomness of Pauli errors and in syndrome measurement. The $\tau_{e}$ spins are nonlocally correlated at timelike direction since all spacelike plaquette interactions $U_{\;p_{s}}$ along the same timelike arrow are all coupled to the same $b_{\Pi (p_{s})}$. Meanwhile, the disorder probability Eq. 5 is also correlated at time direction. Physically this is due to the fact imperfection measurement operator will change the current quantum state, which in turn affects subsequent measurement results. It is evident from the expressions Eqs. 10 and 5 that the nonlocal correlation results from finite $\beta_{0}$, or in other words imperfect initial state preparation.

This SM model is a 3D $Z_{2}$ gauge theory defined on the spacetime lattice coupled to a 2D $Z_{2}$ gauge theory defined on the physical lattice, see Fig. 3. Here $\tau_{e}=\pm 1$ is a spin variable defined on each edge *e* of the spacetime lattice. $U_{p}$ is an ordinary $Z_{2}$ gauge interaction term containing four $\tau_{e}$ operators, $\left[\{\eta_{\;p}\}\right]$ denotes the topological equivalent class represented by $\left\{\eta_{\;p}\right\}$, and $\eta_{p}$ sets the sign of interaction term on each plaquette. Physically, those $\tau_{e}$ operators describe the fluctuation of error strings, since flipping $\tau_{e}$ operator is equivalent to deforming the error strings represented by $\left\{\eta_{\;p}\right\}$. Therefore, the model acquires a local symmetry


(11) $$\eta_{p}\to \eta_{p}\prod_{e\in \partial p}\nu_{e},\;\tau_{e}\to \tau_{e}\nu_{e},\;\nu_{e}=\pm 1,$$

which ensures that topologically equivalent error configurations yield the same partition function.

In order to detect the error threshold, Eq. 5 can be considered as the quenched disorder probability of interaction configuration $\left\{\eta_{p}\right\}$. Then the phase transition point of $\tau_{e}$ spins in the disordered SM model corresponds to the error threshold of the QEC model (28, 32, 37). Note that the 2D gauge model, derived from initial state imperfections (51, 55), and the 3D gauge model, characterizing error string fluctuations (28), couple to form a novel system illuminating imperfect initial states’ effects on QEC.

## Phase structure of the SM model

First, we notice that the SM model has a nonlocal correlation at time direction originates from imperfect initial state preparation, see Fig. 3. If the initial state is well prepared ($\beta_{0}\to \infty$), the code space $\tilde{C}(\beta_{0})$ becomes exactly the toric code subspace. The action of following syndrome measurement operators Eq. 3 on this space yields only a global phase factor and does not change the state itself. In this case, the model reduces to the familiar random plaquette gauge model (RPGM) (28, 60, 61).

In reality, the faulty circuits that produce the imperfect syndrome measurements also provide the imperfect initial state preparations. In this situation, i.e. with finite $\beta_{0}$, the nonlocal timelike correlation will lead to a different phase structure in stark contract to RPGM. In order to detect the phase diagram of this model, we consider the Wilson loop


(12) $$W_{A}=\prod_{\;p\in A}U_{p}=\prod_{e\in \partial A}\tau_{e},$$

which serves as the order parameter for $Z_{2}$ gauge theory. Here *A* is a set of plaquettes representing a surface in spacetime. The product of $U_{p}$’s on surface *A* equals the product of $\tau_{e}$’s on ∂A, which is the boundary of surface *A* and forms a closed loop. In the conventional $Z_{2}$ gauge theory (58, 59) the scaling behavior of Wilson loop expectation values with respect to the loop size distinguishes between the confinement (disordered) phase and the deconfinement (ordered) phase. In the deconfinement phase, it decays exponentially with respect to the perimeter of the loop,


(13) $$W_{A}∼\exp\;(-\mathrm{const}\times |\partial A|),$$

called perimeter law. Here we use |⋅| to denote the cardinal of a set (i.e. the number of the elements of a set). For example |∂A| is the number of edges contained in ∂A. On the other hand, in the confinement phase, the scaling behavior of Wilson loops obeys area law,


(14) $$W_{A}∼\exp\;(-const\times |A_{\min}|),$$

where $A_{\min}$ is the minimal surface enclosed by ∂A. Here we will study the expectation value of Wilson loops in our SM model.

Note that our SM model satisfies a generalized version of Nishimori condition (62), which means that the error rate parameters $\left(\beta_{0},\beta ,K\right)$ in the quenched disorder probability in Eq. 5 are the same as those in the partition function in Eq. 10, respectively. Under this condition, by taking advantage of a local symmetry of the model shown in Eq. 11, we find that (refer to Sec.III of SI (54))


(15) $$[\langle W_{A}\rangle ]=[\langle W_{A}\rangle^{2}].$$

The above equality suggests the absence of gauge glass phase (60), where $\left[\langle W_{A}\rangle \right]$ obeys area law Eq. 14 but $\left[\langle W_{A}\rangle^{2}\right]$ obeys perimeter law Eq. 13. In that case, we only need to concern about the deconfinement–confinement phase transition of $\tau_{e}$’s under Nishimori condition.

We then perform a low-temperature (i.e. $\beta_{0}$, *β* and *K* are sufficiently large, corresponding to small enough physical error rates) expansion (59) for $\left[\langle W_{A}\rangle \right]$. Here, ⟨⋅⟩ denotes the ensemble average with respect to the model Eq. 10 under a specific configuration; and $\left[\cdot ]=\sum_{\left\{\eta_{p}\right\}}P(\{\eta_{p}\})(\cdot \right)$ represents the disorder average over different configurations with respect to the probability Eq. 5. We assume $e^{-\beta_{0}}$, $e^{-\beta }$, and $e^{-K}$ are of the same order and expand $\log\;[\left\langle W_{A}\right\rangle ]$ up to the first nonvanishing order $e^{-4\beta_{0}}$. We obtain the result


(16) $$\begin{array}{rl} & \left[\langle W_{A}\rangle ]≃\exp\;[-4e^{-4\beta_{0}}|\Pi (A)|(N-|\Pi (A)|\right)\\ & \;-\left(e^{-4\beta }+e^{-4K}+4e^{-2\beta -2K}\right)|\partial A{|}_{s}-6e^{-4K}|\partial A{|}_{t}]. \end{array}$$

**Fig. 4.**
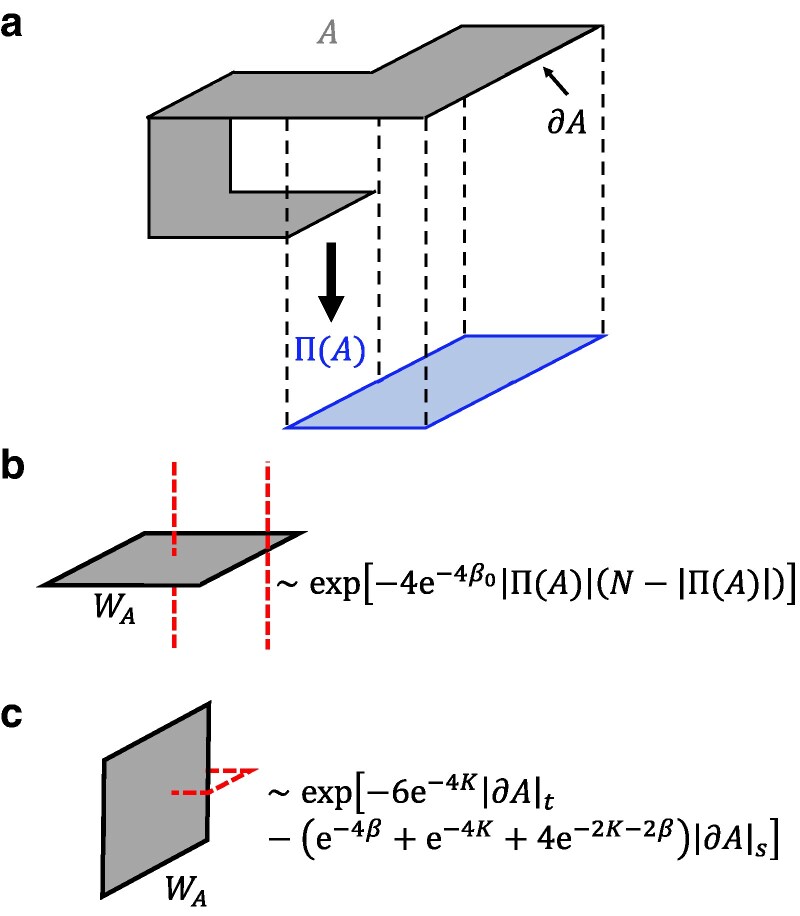
a) Example of a Wilson loop. *A* is a surface in 3D spacetime (upper) and ∂A is its boundary, which is a closed loop. Π(A) is the projection of surface *A* from 3D spacetime to 2D space mod $Z_{2}$ (lower). Specifically, under projection the timelike plaquettes are dropped, and an even number of spacelike plaquettes at the same space position also vanishes. The only remaining plaquettes are at the spatial locations that originally have odd numbers of spacelike plaquettes. In (b) and (c), the black lines represent Wilson loops and the red dashed lines are examples of topologically trivial error strings created by $\tau_{e}$ fluctuation. b) A spacelike Wilson loop. In a large enough system it decays exponentially with respect to the area for any finite temperature. For a large region *A*, the areal decay will be much faster than the perimetric decay, so the first term in Eq. 16 dominates. This behavior is contributed by the fluctuation of infinite long timelike error strings which are able to appear at any space position (see Sec.SIV of SI (54)). c) A timelike Wilson loop. Under a low temperature, it decays exponentially with respect to the perimeter. Note that both timelike edges and spacelike edges are contained in the boundary of a timelike region, determining the $|\partial A{|}_{t}$ term and $|\partial A{|}_{s}$ term, respectively.

Here *Π* is defined as projection from 3D spacetime to 2D space mod $Z_{2}$, illustrated in Fig. 4a. $|\partial A{|}_{s}$ ($|\partial A{|}_{t}$) denotes the spacelike (timelike) edges $e_{s}$ ($e_{t}$) contained in ∂A. The derivation of the expansion is lengthy and refer to Sec.SIV of SI (54) for details.

**Fig. 5.**
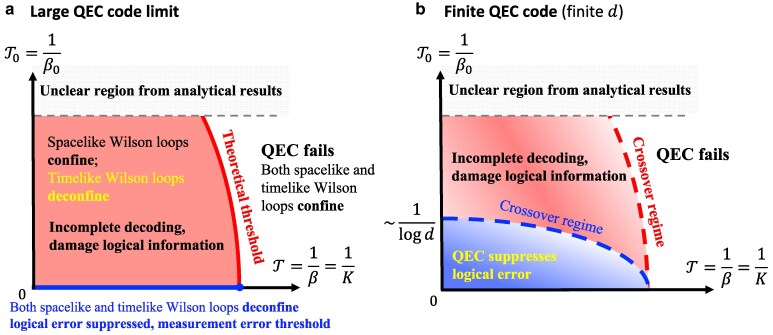
a) Our estimation of the phase structure in the thermodynamic limit T→+∞ and N→+∞ while setting T=1/β=1/K and $T_{0}=1/\beta_{0}$. Above the theoretical threshold QEC fails due to noncontractible logical Pauli errors. Below the theoretical threshold and above the measurement error threshold, noncontractible logical Pauli errors are suppressed. However, measurement errors are still unidentifiable through decoding a finite error history and will be confounded with Pauli errors. Note that the T axis where $T_{0}=0$ represents the RPGM. While the theoretical threshold intersect with the T axis at the common RPGM phase transition point (28), we are not sure yet whether it intersect with the $T_{0}$ axis. Some details at higher temperatures still requires further investigation. b) The phase diagram while fixing a finite code distance *d*. The phase transitions (thresholds) in Fig. 5a are smoothed into crossovers due to finite-size effect. Especially, the measurement error threshold becomes a finite-temperature crossover (lower dashed line), leading to a parameter region with a finite area (lower region) that effectively suppresses logical errors. The parameters in this region should satisfy either Eq. 17 or Eq. 18 such that the nonlocal measurement errors will not be a problem. Specifically, the crossover condition $T_{0}∼1/\log\;d$ near the $T_{0}$ axis is derived from Eq. 17. (However, Eq. 17 or Eq. 18 are only approximate expressions valid in the low-temperature limit. The precise value of this crossover still requires further investigation.) Increasing *d*, this region becomes smaller and smaller and eventually sticks to the T axis. Above the crossover regime of measurement threshold in the light red region, the effect of nonlocal measurement errors on the QEC becomes nonnegligible. As for the SM model side, the blue crossover detects the confinement of spacelike Wilson loops while the red crossover detects the confinement of timelike Wilson loops.

Equation 16 provides us with insights into the phase structure. As we have mentioned, the Wilson loops have anisotropic scaling behavior at low temperatures. A pure timelike Wilson loop $W_{A}$ which contains only timelike plaquettes is shown in Fig. 4c. It deconfines and decays exponentially with respect to perimeter as in a conventional 3D $Z_{2}$ lattice gauge theory under low temperature. Meanwhile, a pure spacelike Wilson loop is shown in Fig. 4b. For large enough system size *N*, its areal decay is faster than the perimetric decay, so the first term in Eq. 16 dominates and $\left[\left\langle W_{A}\right\rangle \right]$ confines as long as the temperature is finite. We notice that no matter how low the nonzero temperature is, the confinement is always maintained. In addition, a sufficiently high temperature should always drive the system into a completely disordered phase, which will confine all Wilson loops. Thus, the spacelike Wilson loops confine at any finite temperature (or error rate). In comparison, if the initial state is ideally prepared ($\beta_{0}=+\infty$, corresponds to RPGM), the Wilson loops will acquire an isotropic scaling behavior, i.e. both the spacelike and timelike Wilson loops will exhibit perimetric decay at the low-temperature phase and areal decay at the high-temperature phase (28). The qualitative phase diagram is summarized in Fig. 5a.

## Impact on QEC

Following the mapping of our QEC model to an SM model, we now examine its implications on QEC performance and the threshold theorem.

### Behavior in an infinite size system

First of all, recognizing the existence of a confinement transition point concerning timelike Wilson loops in the limit T→+∞ and N→+∞, we ascertain that it also separates different behaviors of logical error rate. In fact, we find that the logical errors associated with spacelike noncontractible loops are suppressed at the low-temperature phase, indicating a transition we refer to as the theoretical threshold. In our derivation of low-temperature expansion (Sec.SIV of SI (54)), we find that:

1. The decay in area law for spacelike Wilson loops is attributed to the emergence of nonlocal timelike error strings as depicted in Fig. 4b.
2. At sufficiently low temperatures, nonlocal timelike strings and local error loops manifest with relative independence, resulting in the confinement of local error loops, thereby preventing their extension to arbitrary lengths.

Specifically, this implies that fluctuating error strings are constrained (or unable to extend indefinitely) in their spatial extension, leading to a negligible error rate for spacelike noncontractible loops or logical Pauli operators. By examining the domain wall free energy cost (28) associated with these noncontractible loops, it is found to be proportionate to the code distance *d* within the red phase as shown in Fig. 5a. By increasing the temperature, a transition point is anticipated where timelike Wilson loops become confined, concurrently with the proliferation of spacelike noncontractible error strings. However, this threshold, which will be elucidated subsequently, does not encompass the correctability of measurement errors. Above this theoretical threshold, QEC is deemed ineffective, as evidenced by a persistent and finite logical error rate that is not ameliorated by enlarging the system size, particularly within the red phase depicted in Fig. 5a.

Recall that confinement of spacelike Wilson loops is observed even below the theoretical threshold due to the fluctuation of timelike nonlocal error strings (Fig. 4b). Although those timelike error strings do not directly relate to logical Pauli operators, they signify the proliferation of measurement errors. Those nonlocal measurement errors (extending the whole time interval [0,T] for any finite *T*) become indistinguishable from local Pauli errors and thus damage the decoding process. For example, the true syndrome of a single Pauli error string will be two nonlocal timelike strings starting from its boundary points. But in the meantime, the syndrome can be generated by nonlocal measurement errors, which may occur with a probability comparable to that of the Pauli string and are impervious to suppression by large *T* or large *d*. The consequence is that the decoder might mix up these two situations with a finite probability and inappropriately omit the correction of the aforementioned Pauli error. If this Pauli error anticommutes with logical *Z* operators, the logical information will be damaged. Note that in this case, the defects caused by faulty measurement and Pauli error complements each other in the physical states, so the error cannot be detected even when applying more error correction cycles. In the low-temperature limit, we expect the logical error rate (measured with worst-case infidelity) to be proportional to the code distance, see Sec.SV of SI (54). Given that the nonlocal timelike error strings consistently perforate the entire lattice, the choice of *T* in reality for decoding is inconsequential. Hence, the logical error rate is also not suppressed by increasing *T*. These results indicate the loss of quantum information.

Henceforth, our investigation elucidates crucial findings: Timelike nonlocal measurement errors and local Pauli errors together constitute a new mechanism that causes logical errors, and the probability of those events cannot be suppressed by large *T* or *d*. So when $\beta_{0}$ is finite, the QEC system behaves like it is above the true error threshold. Notice that the above mechanism is not explicit in the infinite time decoding scenario, since the nonlocal timelike syndromes are imagined to be connected by error strings at both the t=+∞ and t=−∞ ends, constituting complete world lines. This is not true in a realistic decoding process, because only finite error history can be taken into consideration.

This is in stark contrast to the case with only stochastic errors (28), where the perimeter law for both the spacelike and timelike Wilson loops guarantees the achievement of an effective error correction with a finite error history. Furthermore, it is worth noting that the RPGM (28) manifests an isotropic characteristic, i.e. the simultaneous deconfinement and confinement of the spacelike and timelike Wilson loops, respectively, hence, obviating the need for distinct analyses of various error types.

In our case, the inability to correct measurement errors is caused by the finite value of $\beta_{0}$, i.e. the imperfection of initial state preparation; so we refer to $\beta_{0}\to +\infty$ as the measurement error threshold of our error model. Setting β=K, a sketch of the phase diagram is shown in Fig. 5a. In addition, a comparison between our error model and that with only stochastic errors (28) is shown in Table 1.

**Table 1.** Comparison between our case and the case with the stochastic measurement error model (28).

	Our case	The case of Ref. (28)
Noise properties	1) Coherent errors on entanglement gates of stabilizer measurement circuit (imperfect measurement);	1) Stochastic measurement errors of stabilizer measurement outcomes;
	2) Stochastic Pauli errors on physical qubits.	2) Stochastic Pauli errors on physical qubits.
Initial state	Affected by imperfect measurement during preparation (characterized by $\beta_{0}$).	Ideal toric code state.
Error correction protocol	Multiround syndrome measurement (number of rounds: *T*) and maximum-likelihood decoder.	
SM mapping	3D $Z_{2}$ gauge model coupled to a 2D $Z_{2}$ gauge model with quenched disorder.	3D RPGM under Nishimori condition.
Phase structure of SM model	When $\beta_{0}\to +\infty$ (ideal initial state), the SM model reduces to RPGM.	Low-temperature phase (below threshold): all Wilson loops deconfine.
	For finite $\beta_{0}$ (imperfect initial state):	High-temperature phase (above threshold): all Wilson loops confine.
	– Low-temperature phase (below theoretical threshold): timelike Wilson loops deconfine, spacelike loops confine.	
	– High-temperature phase (above threshold): all Wilson loops confine.	
Error correction performance	$\beta_{0}\to +\infty$ : equivalent to the model in Ref. (28).	Low-temperature phase (below theoretical threshold): QEC success probability enlarged by increasing *N* and *T* ($T\gg \sqrt{N}$ required; QEC always fails when T=O(1))
	Finite $\beta_{0}$:	High-temperature phase (above theoretical threshold): QEC fails.
	– Low-temperature phase (below theoretical threshold): unidentifiable errors degrade QEC performance (even for large *T*).	
	– High-temperature phase (above theoretical threshold): QEC fails (noncontractible error loops).	

Note that although the measurement noises of the two models are different at the physical level, their error correction properties and corresponding SM models will become equivalent when we set the initial state in our model to be ideal ($\beta_{0}\to +\infty$).

For completeness, we scrutinize the phase structure in various limits. Notice that the red phase in Fig. 5a signifies the proliferation of measurement error strings, but logical errors result from a combination of measurement errors and Pauli errors. In the limit K→+∞ (keeping $\beta_{0}$ and *β* finite), the QEC protocol trivially succeeds as there will be no Pauli errors. But as long as *K* is finite, the logical error rate is not suppressed by the system size due to the indistinguishability between measurement errors and Pauli errors. In the scenario where β→+∞ but *K* and $\beta_{0}$ are finite, the nonlocal measurement errors are still present and cannot be decoded when intertwined with Pauli errors. As for the $\beta \to_{0}+\infty$ limit, it reduces to the RPGM as mentioned before.

### Finite-size effect

For a small code with limited system size *N*, we infer from Eq. 16 that the above problem might be circumvented with the limit


(17) $$d\ll e^{\beta_{0}},$$

or


(18) $$d\ll [e^{4\beta_{0}}(e^{-4\beta }+e^{-4K}+4e^{-2\beta -2K}){]}^{1/3}.$$

Here *d* is the code distance and $N=d^{2}$. The first bound is derived by assuming the areal term in Eq. 16 is negligible


(19) $$e^{-4\beta_{0}}|\Pi (A)|(d^{2}-|\Pi (A)|)\ll 1,$$

for all spacelike Wilson loops *A*. The left-hand-side maximizes when *A* is half the size of the spatial lattice $|\Pi (A)|=d^{2}/2$. Substituting $|\Pi (A)|=d^{2}/2$ into the above expression, we have


(20) $$e^{-4\beta_{0}}d^{4}/4\ll 1,$$

which is equivalent to Eq. 17 ignoring a constant factor. Physically, Eq. 17 is interpreted as the probability of the appearance of nonlocal measurement errors on the whole system is ignorable. The second bound is derived by considering the case when the perimetric decay is faster than the areal decay in Eq. 16 for a spacelike Wilson loop,


(21) $$\begin{array}{rl} & e^{-4\beta_{0}}|\Pi (A)|(d^{2}-|\Pi (A)|)\\ & \;\ll \left(e^{-4\beta }+e^{-4K}+4e^{-2\beta -2K}\right)|\partial A{|}_{s}. \end{array}$$

Still we require *A* to be half of the spatial lattice, $|\Pi (A)|∼d^{2}$ and $|\partial A{|}_{s}∼d$. Thus, we have


(22) $$e^{-4\beta_{0}}d^{4}\ll \left(e^{-4\beta }+e^{-4K}+4e^{-2\beta -2K}\right)d,$$

which leads to Eq. 18. Physically, Eq. 18 is interpreted as the influence of nonlocal error strings is not significant compared to other local error strings. If either of these two bounds is satisfied, we anticipate that the ability of our QEC procedure to detect measurement errors will be similar to that of Ref. (28). To do so, the imperfection of initial state preparation must be negligible or much smaller than the syndrome measurement imperfection and Pauli error rate. Even then the code distance is still upper bounded if we fix the error parameters $\beta_{0}$, *β*, and *K*. Usually, when performing QEC, we anticipate increasing code distance to suppress the logical error rate (29). However, for the important error problem considered here, Eqs. 17 and 18 form bounds that prevent the code from scaling up. Equivalently if we fix *d* and vary the error parameters, we obtain the phase diagram in Fig. 5b. It is noteworthy that the region enabling pragmatic error correction only experiences a gradual reduction as increasing the code distance, i.e. ∼1/logd, which is not excessively frustrating. In Sec.SVII of SI (54), we describe a preparation procedure with the multiround measurement protocols that maintains decodability in small finite code systems.

## Relation to a realistic measurement circuit

In fact, the circuit shown in Fig. 1b, which contains only two-qubit gates rather than a five-qubit evolution in our simple model, is more realistic, and it is expected to have worse performance while suffering from coherent noise on entanglement gates. Yang & Liu (50) discussed an imperfect measurement model that mimics the behavior of superconducting quantum computation systems. The *CNOT* gate is divided into a *CZ* gate and two Hadamard gates, CNOT=H(CZ)H where *H* is the Hadamard gate acting on the target qubit (ancilla qubit in our setup). Each *CZ* gate is implemented by a time evolution


(23) $$U=\exp\;\left[-i\frac{t}{4}\left(s^{z}⊗\sigma_{i}^{z}-s^{z}⊗I-I⊗\sigma_{i}^{z}+I⊗I\right)\right]$$

Here *s* labels the ancilla qubit and $\sigma_{i}$, i=1,2,3,4 labels the four data qubits. It recovers the *CZ* gate when t=π. Assume the final ancilla measurement has an outcome s=±1, the corresponding action on data qubits is


(24) $$\begin{array}{rl} M_{s} & =|s\rangle H\exp\;\left[-i\frac{t}{4}\sum_{i}\left(s^{z}⊗\sigma_{i}^{z}-s^{z}-\sigma_{i}^{z}+I\right)\right]H|0\rangle \\ & \;=\frac{1}{2}\left(1+se^{-i2t}\cos^{4}\;\frac{t}{2}+se^{-i2t}\sin^{4}\;\frac{t}{2}B_{\;p_{0}}\right)\\ & \;\times (1-\frac{ise^{i2t}\sin\;\frac{t}{2}\cos^{3}\;\frac{t}{2}+i\sin\;\frac{t}{2}\cos^{7}\;\frac{t}{2}+i\sin^{7}\;\frac{t}{2}\cos\;\frac{t}{2}}{{\left(se^{i2t}+\cos^{4}\;\frac{t}{2}\right)}^{2}-\sin^{8}\;\frac{t}{2}}\sum_{i}\sigma_{i}^{z}\\ & \;-\frac{\sin^{2}\;\frac{t}{2}\cos^{2}\;\frac{t}{2}}{se^{i2t}+\cos^{4}\;\frac{t}{2}+\sin^{4}\;\frac{t}{2}}\sum_{i<j}\sigma_{i}^{z}\sigma_{j}^{z}\\ & \;+\frac{ise^{i2t}\sin^{3}\;\frac{t}{2}\cos\;\frac{t}{2}+i\sin^{3}\;\frac{t}{2}\cos^{5}\;\frac{t}{2}+i\sin^{5}\;\frac{t}{2}\cos^{3}\;\frac{t}{2}}{{\left(se^{i2t}+\cos^{4}\;\frac{t}{2}\right)}^{2}-\sin^{8}\;\frac{t}{2}}\sum_{i<j<k}\sigma_{i}^{z}\sigma_{j}^{z}\sigma_{k}^{z}). \end{array}$$

When t=π, one may check that the above expression reduces to the correct projection $(I+sB_{\;p_{0}})/2$. When t≠π, $M_{s}$ stands for an imperfect measurement operator.

To understand the effect of the realistic error model Eq. 24, we compare it with the simplified model Eq. 3. As a picture of how decoding fails for imperfect initial states, recall that those states contain superpositions of defects (stabilizer generator =−1 components) whose amplitudes do not decay with the distance between defects. The consequence is that the failure probability of decoding will not decay with code distance. We notice that in its expression the first factor $1+se^{-i2t}\cos^{4}\;(t/2)+se^{-i2t}\sin^{4}\;(t/2)B_{\;p_{0}}$ is similar to the imperfect measurement operator discussed in Eq. 3  $\exp\;(\beta s_{\;p_{0}}B_{\;p_{0}}/2)=\cosh\;(\beta /2)+s_{\;p_{0}}\sinh\;(\beta /2)B_{\;p_{0}}$. Recall that in the simple model depicted in Fig. 1c, the absence of a finite measurement error threshold is attributed to the superposition of defects with $B_{\;p_{0}}=-1$. The amplitudes of these defects remain constant regardless of their spatial separation and depend solely on their quantity. In contrast, the realistic model illustrated in Fig. 1b incorporates the term $\exp\;(\beta s_{\;p_{0}}B_{\;p_{0}}/2)$. This inclusion leads to superpositions analogous to those in the simple model, thereby suggesting that the issue of lacking a finite threshold persists in the realistic scenario. Furthermore, there is an additional factor, which can be viewed as coherent errors appearing on data qubits. These coherent will further lead to $A_{v}=-1$ defects in the initial code state (see Sec. SVIII of SI). So while discussing error correction, aside from the previously discussed consequences, coherent errors are likely to inflict greater damage on the error correction process, resulting in deteriorated performance. We also notice that under this realistic measurement model, if we define logical states by applying logical operators to the imperfect initial state, those states will not be orthogonal to each other, making it much harder to solve exactly. Finally, we stress that the preceding discussion concerning the realistic model relies on model-based calculations and partially qualitative reasoning. A rigorous mathematical proof for general cases is essential and merits comprehensive investigation in future studies.

For a small finite code, when considering the application of the multiround measurement preparation protocol to specific logical states within the Pauli basis, its effectiveness is brought into question for more realistic measurement circuits. In this scenario, the implementation of the $A_{v_{0}}$ measurement is necessitated for the correction of the *Z* coherent error strings induced by the $B_{\;p_{0}}$ measurement. Given that these two distinct types of imperfect stabilizer measurement operators are noncommutative, their resulting measurement outcomes are inherently interdependent. Future investigations could benefit from exploring a hybrid decoding approach that combines both $A_{v_{0}}$ and $B_{\;p_{0}}$ stabilizer measurement data.

## Discussions

We study the performance of toric code QEC under the influence of various important error types, including imperfect measurement circuits for both preparation and error detection, as well as the qubit-level stochastic Pauli noise. We establish a connection between the corresponding QEC performance and a novel SM model. Our SM analysis reveals the mechanism of how the imperfect initial code states affect the following error correction cycle. We emphasize that the code state preparation requires special attention, which was rarely discussed in previous theoretical studies of QEC error thresholds. A naive preparation protocol could destroy the ability to shield quantum information from local Pauli perturbations. Although focusing on toric code in the calculations, we expect our results to hold for general surface codes in sufficiently large sizes. The terminology “surface code” refers to the stabilizer code Eq. 1 in various boundary conditions, with toric code a special case. We expect that the spatial boundary conditions are not expected to affect the qualitative properties of the SM model in the thermodynamic limit. In addition, we mention that QEC code states could be prepared by pure unitaries instead of measurements (21, 63, 64). However in large systems, the typical circuit depth of unitary preparation is larger than measurement preparation in the ideal case, and its resilience against coherent deviations could be even worse.

Before closure, it is important to highlight that our results indicate a different impact based on the scale of quantum codes. Specifically, smaller quantum codes, typically situated within the 1/logd threshold, still exhibit resilience against the outlined challenges (Sec.SVII of SI (54)), thereby maintaining robust QEC capabilities. Current small-scale experiments of surface code QEC (6, 17, 18, 20, 21, 24) are believed to stay in the effectively correctable region such that they provides positive results. Conversely, our findings hold considerable significance for larger-scale systems, which are central to the realization of application-level fault-tolerant quantum computing. As the scale of the system expands, the criticality of code space preparation must be significantly intensified, accompanied by an increased consumption of resources. This observation emphasizes the crucial need for a focus on refining state preparation protocols, a measure essential to safeguarding the efficacy and feasibility of QEC for advanced fault-tolerant quantum computations.

## Supplementary Material

pgaf063_Supplementary_Data

## Data Availability

Data sharing is not applicable to this article, as no datasets were generated or analyzed during the current study.

## Preprints

This manuscript was posted on a preprint: [10.48550/arXiv.2301.12859].
