# Supplementary material for: Vulnerability of fault-tolerant topological quantum error correction to quantum deviations in code space
Source: PNAS Nexus. 2025 Feb 25;4(3):pgaf063. doi: 10.1093/pnasnexus/pgaf063 (PMC11897702; doi:10.1093/pnasnexus/pgaf063)
Supplement: pgaf063_Supplementary_Data [file pgaf063_supplementary_data.pdf]

Vulnerability of fault-tolerant topological quantum error correction to quantum deviations in code space

In this supplemental information, we provide details about derivations of the results in the main text. In Sec. SI we discuss some properties about code space when initial state preparation suffers from imperfection measurement. In Sec. SII we derive the SM mapping explicitly. In Sec. SIII we talk about the consequence of local symmetry under the Nishmori condition. In Sec. SIV we derive the low-temperature expansion of the Wilson loop. In Sec. SV we examine the behavior of logical error rate in the low-error-rate limit. In Sec. SVI we have a simple discussion on the case where the coherent deviation in the imperfect measurement model is IID instead of fixed to a specific value. In Sec. SVII, we provide a code state preparation procedure to stay in the decodable region for finite codes. In Sec. SVIII, we discuss the imperfect code state under the assumption of the realistic measurement circuit.

SI. CODE SUBSPACE UNDER IMPERFECT MEASUREMENT

Here we discuss the imperfect code subspace under the presence of coherent errors on entanglement gates during state preparation. Starting with a product state $\bigotimes_{e_0} |+\rangle_{e_0}$, we obtain the (unnormalized) state $M_{\{s_{p_0}\}} \bigotimes_{e_0} |+\rangle_{e_0}$ with probability

$$P(\{s_{p_0}\}) = \bigotimes_{e_0} \langle +|_{e_0} E_{\{s_{p_0}\}} \bigotimes_{e_0} |+\rangle_{e_0} = \frac{1}{(8\cosh\beta)^N} \mathcal{Z}_{\{s_{p_0}\}}, \tag{S1}$$

$$\mathcal{Z}_{\{s_{p_0}\}} = \sum_{\{\sigma_{e_0}\}} \exp\left[\beta \sum_{p_0} s_{p_0} b_{p_0}\right], b_{p_0} = \prod_{e_0 \in \partial p_0} \sigma_{e_0}, \tag{S2}$$

where $M_{\{s_{p_0}\}}$ is the imperfect measurement operator

$$M_{\{s_{p_0}\}} = \frac{1}{(\sqrt{2}\cosh\beta)^N} \exp\left[\frac{1}{2}\beta \sum_{p_0} s_{p_0} B_{p_0}\right], \tag{S3}$$

and $E_{\{s_{p_0}\}} = M^\dagger_{\{s_{p_0}\}} M_{\{s_{p_0}\}}$ is the corresponding POVM operator. By expanding $\bigotimes_{e_0} |+\rangle_{e_0}$ under computational basis $\bigotimes_{e_0} |+\rangle_{e_0} = (1/2^N) \sum_{\{\sigma_{e_0}\}} \bigotimes_{e_0} |\sigma_{e_0}\rangle$, where $\sigma_{e_0} = \pm 1$ is the eigenvalue of Pauli operator Z_{e_0}, it can be shown that $P(\{s_{p_0}\})$ is proportional to the partition function of a 2-d $\mathbb{Z}_2$ lattice gauge theory $\mathcal{Z}_{\{s_{p_0}\}}$ [1, 2], which is a useful method to dealing with the post-measurement state and is helpful to our following derivations. Note that 2-d $\mathbb{Z}_2$ lattice gauge theory is exact-solvable [3]. By noticing that the Boltzmann weight only depends on the value of b_{p_0}, we rewrite the summation of spin configurations as a summation of b_{p_0} configurations together with 1-form symmetry operations

$$\sum_{\{\sigma_{e_0}\}} = \sum_{\{b_{p_0}\}} \delta_{\prod_{p_0} b_{p_0}, 1} \sum_{\text{dual loop}} . \tag{S4}$$

Here $\delta_{\prod_{p_0} b_{p_0},1}$ means that the product of all b_{p_0} must equal to 1, since the lattice is embedded in a torus surface. We may check the degree of freedoms involved in the summation $2^{2N} = 2^N/2 \times 2^{N+1}$. Thus we have

$$\begin{aligned}\mathcal{Z}_{\{s_{p_0}\}} &= \sum_{\{b_{p_0}\}} \delta_{\prod_{p_0} b_{p_0},1} \sum_{\text{dual loop}} \exp\left[\beta \sum_{p_0} s_{p_0} b_{p_0}\right] \\ &= 2^{N+1} \sum_{\{b_{p_0}\}} \frac{1+\prod_{p_0} b_{p_0}}{2} \exp\left[\beta \sum_{p_0} s_{p_0} b_{p_0}\right] \\ &= 2^N \prod_p \left(\sum_{b_{p_0}} \exp\left[\beta s_{p_0} b_{p_0}\right] + \sum_{b_{p_0}} b_{p_0} \exp\left[\beta s_{p_0} b_{p_0}\right]\right) \\ &= 2^N (2\cosh\beta)^N + 2^N (2\sinh\beta)^N \prod_{p_0} s_{p_0},\end{aligned} \tag{S5}$$

and hence we have the probability of measurement outcomes

$$P(\{s_{p_0}\}) = \frac{1+(\tanh\beta)^N \prod_p s_{p_0}}{2^N}. \tag{S6}$$

Note that $\prod_{p_0} s_{p_0}$ is the parity of $\{s_{p_0}\}$ configuration.

From the above discussion, it can be seen that we might get different states with respect to the ancilla measurement outcome $\{s_{p_0}\}$. But we will fix the initial state as $M_{\{+\}} \bigotimes_{e_0} |+\rangle_{e_0}$, where we assumed the measurement outcomes of ancilla qubits are all $+1$. For other measurement outcomes $\{s_{p_0}\}$, we can redefine the sign of our stabilizers $B_{p_0} \to s_{p_0} B_{p_0}$ in order that the following discussions still apply. The only thing we should take into consideration is the parity of outcome $\{s_{p_0}\}$ because under the redefinition $\prod_{p_0} B_{p_0} = 1 \to \prod_{p_0} B_{p_0} = \prod_s s_{p_0}$ and we will get -1 for odd parity. In the odd parity case, the defects on the lattice cannot be paired up. We cannot redefine an odd number of B_{p_0}'s and still satisfy the constrain $\prod_{p_0} B_{p_0} = 1$, and start QEC with a state where defects already present won't be a benefit to the performance. Hence we ignore odd parity outcomes. We might consider it as a post-selection procedure, which chooses the even parity result that occurs with probability $P_+ = (1 + (\tanh\beta)^N)/2$, see Eq. (S6). We find that $1/2 \le P_+ \le 1$ for $0 \le \beta \le +\infty$. Note that this probability is close to 1 when β is sufficiently large, so the post-selection procedure is reasonable for experimental consideration.

Till now we have only obtained one logical state. What about other states in the imperfect code subspace? The problem is that the matrix rank of $M_{\{s_{p_0}\}}$ is 2^{2N}, which means that if we view it as a map defined on the whole 2^{2N} dimensional Hilbert space of physical qubits, $M_{\{s_{p_0}\}} : \mathcal{H} \to \mathcal{H}$, then we find that $Im(M_{\{s_{p_0}\}}) = \mathcal{H}$. This is different from the projective measurement case that $P_{\{s_{p_0}\}} = \prod_{p_0} (I + s_{p_0} B_{p_0})/2$ projects any states into the $B_{p_0} = s_{p_0}$ subspace. That is why we define the code space using logical operators in analogy to experimental setups. In summary, our four logical basis states are defined as

$$\begin{aligned}|\widetilde{++}\rangle &= \frac{M_{\{+\}} \bigotimes_{e_0} |+\rangle_{e_0}}{\sqrt{\bigotimes_{e_0} \langle+|_{e_0} M_{\{+\}}^\dagger M_{\{+\}} \bigotimes_{e_0} |+\rangle_{e_0}}}, \\ |\widetilde{-+}\rangle &= Z_{l_1} |\widetilde{++}\rangle, \quad |\widetilde{+-}\rangle = Z_{l_2} |\widetilde{++}\rangle, \quad |\widetilde{--}\rangle = Z_{l_1} Z_{L_2} |\widetilde{++}\rangle,\end{aligned} \tag{S7}$$

Notice that $|\widetilde{++}\rangle$ state correspond to the model

$$\mathcal{Z}_{\{+\}} = \sum_{\{\sigma_{e_0}\}} \exp\left[\beta \sum_{p_0} b_{p_0}\right]. \tag{S8}$$

Given the above imperfect logical states, the expectation value of Pauli Z operators can be computed through the classical SM model [1, 2]. We denote $\langle\cdot\rangle_{\{+\}}^q$ as the expectation value of the post-measurement state $|\widetilde{++}\rangle$, then

$$\left\langle \prod_{e_0 \in c_0} Z_{e_0} \right\rangle_{\{+\}}^q = \left\langle \prod_{e_0 \in c_0} \sigma_{e_0} \right\rangle_{\{+\}}^c. \tag{S9}$$

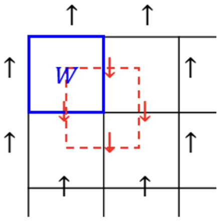

FIG. S1. 2-d $\mathbb{Z}_2$ lattice gauge theory. The classical spins σ_{e_0} are associated with each edge. Both the Hamiltonian and Wilson loop (blue solid loop) are invariant under 1-form symmetry operation (red dashed loop) which flips the spins on a dual loop.

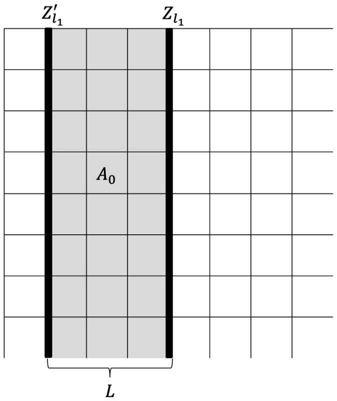

FIG. S2. Different definitions of logical Pauli Z operator. Under imperfect measurement preparation, the two logical states defined by them are different. The fidelity between these two states relates to the expectation value of a Wilson loop observable constituted by these two non-contractible loops.

where we denote c_0 as a set of several edges and $\langle\cdot\rangle^c_{\{+\}}$ as the expectation value of the classical model $\mathcal{Z}_{\{+\}}$.

2-d $\mathbb{Z}_2$ lattice gauge theory possesses 1-form symmetry [3]. As in Fig. S1, if we flip all the spins along a dual loop, it does not change the value of b_{p_0} on any plaquette p_0, hence does not change the partition function, i.e. Eq. (S2).

For 2-d $\mathbb{Z}_2$ lattice gauge theory, Elitzur's theorem [3, 4] states that the expectation value of any observable that varies under 1-form symmetry operation vanishes (even under the presence of infinitesimal source term). For example, consider the overlap between two logical states

$$\langle\widetilde{-+}|\widetilde{++}\rangle = \langle\widetilde{++}|\, Z_{l_1}\, |\widetilde{++}\rangle = \langle Z_{l_1}\rangle^q_{\{+\}} = \left\langle \prod_{e_0\in l_1} \sigma_{e_0} \right\rangle^c_{\{+\}}. \tag{S10}$$

The operator $\prod_{e_0\in l_1}\sigma_{e_0}$ is the product σ_{e_0} spins that win around the non-contractible loop l_1, and it changes sign under the 1-form symmetry operation that flips the spins on a dual non-contractible loop l_1^* that intersect with l_1. Consequently, we find that $\langle\widetilde{-+}|\widetilde{++}\rangle = 0$. Note that this result can also be checked through explicit calculation since $\mathcal{Z}_{\{+\}}$ acquires an exact solution. Similarly, we find that all the four states $\{|\widetilde{++}\rangle, |\widetilde{-+}\rangle, |\widetilde{+-}\rangle, |\widetilde{--}\rangle\}$ are orthogonal to each other. Therefore, they form an orthonormal basis of a 4 dimensional subspace. This justifies our definition of the imperfect code subspace

$$\widetilde{\mathcal{C}}(\beta) = \text{span}\{|\widetilde{++}\rangle, |\widetilde{-+}\rangle, |\widetilde{+-}\rangle, |\widetilde{--}\rangle\} \tag{S11}$$

What is more, these four states are still eigenstates of logical X operators. Since $X_{l_1^*}, X_{l_2^*}$ commute with imperfect measurement operator $M_{\{+\}}$ and act as 1 on the initial product state, we find that $X_{l_1^*}|\widetilde{++}\rangle = |\widetilde{++}\rangle, X_{l_2^*}|\widetilde{++}\rangle = |\widetilde{++}\rangle$. The act on the other three states is determined by the commutation relation between logical X and logical Z. Note that these properties are not universal for imperfect measurement but depend on the specific measurement protocol.

Here we point out that, unlike the projective measurement case, $\widetilde{\mathcal{C}}$ depends on the choice of logical operators on the lattice. For example, consider a different choice of logical Z operator Z'_{l_1} as in Fig. S2. We can calculate the fidelity

between $|\widetilde{-+}\rangle$ and the new state defined by the new logical operator $|\widetilde{-+}\rangle' = Z'_{l_1}|\widetilde{++}\rangle$:

$$\langle\widetilde{-+}|\widetilde{-+}\rangle' = \langle\widetilde{++}|\, Z_{l_1} Z'_{l_1}\, |\widetilde{++}\rangle = \langle Z_{l_1} Z'_{l_1}\rangle^{q}_{\{+\}} \tag{S12}$$

Note that $Z_{l_1} Z'_{l_1}$ forms a Wilson loop that could be written as the boundary of a region A. For the classical model, Wilson loops are 1-form symmetry invariant observables and acquire non-zero expectation values. For example, consider the Wilson loop

$$W_{A_0} = \prod_{p_0\in A_0} b_{p_0} = \prod_{e_0\in\partial A_0} \sigma_{e_0}, \tag{S13}$$

where A_0 is a 2-d region (a set of plaquettes) and ∂A_0 is the set of edges at the boundary of A_0. Its expectation value can be evaluated similarly as the partition function, which leads to

$$\langle W_{A_0}\rangle^{c}_{\{+\}} = \frac{(\tanh\beta)^{|A_0|} + (\tanh\beta)^{N-|A_0|}}{1+(\tanh\beta)^N}. \tag{S14}$$

With the help of Eq. (S14), we obtain:

$$\langle\widetilde{-+}|\widetilde{-+}\rangle' = \frac{(\tanh\beta)^{dL} + (\tanh\beta)^{N-dL}}{1+(\tanh\beta)^N} \tag{S15}$$

which is smaller than 1 for finite β, implies that the two states are different.

In addition, notice that we can take the product of the eigenstates of $N-1$ B_{p_0} operators, $N-1$ A_{v_0} operators, and 2 logical operators $X_{l_1^*}$ and $X_{l_2^*}$ to form a complete basis of the whole Hilbert space of physical qubits. Under this basis the product $|+\rangle$ state can be written as

$$\bigotimes_{e_0} |+\rangle_{e_0} = \bigotimes\nolimits'_{p_0} \sum_{b_{p_0}=\pm} |B_{p_0} = b_{p_0}\rangle \bigotimes\nolimits'_{v_0} |A_{v_0} = +\rangle \bigotimes |X_{l_1^*} = +\rangle \bigotimes |X_{l_2^*} = +\rangle\,, \tag{S16}$$

Here the prime on the product symbol means that a chosen plaquette or vertex is excluded to satisfy the global constrain $\prod_{p_0} B_{p_0} = \prod_{v_0} A_{v_0} = I$. This formula is verified as follows. Since the action of A_{v_0}'s and logical X operators on $\bigotimes_{e_0}|+\rangle_{e_0}$ all yield $+1$, the state must be the $+1$ eigenstate of these operators. Besides, assume the excluded plaquette is f_0. Consider a Pauli X string $X_{(f_0\to q_0)}$ that starts at f_0 and ends at some other plaquette q_0. Since $X_{(f_0\to q_0)}$ commutes with all $N-1$ A_{v_0} operators, logical X operators and $N-2$ B_{p_0}'s with $p_0 \neq q_0, f_0$, it only acts on the factor $|B_{q_0} = \pm\rangle$ under the above basis. Note that $X_{(f_0\to q_0)}$ anti-commutes with B_{q_0}, so $X_{(f_0\to q_0)}|B_{q_0} = \pm\rangle = |B_{q_0} = \mp\rangle$. Since $X_{(f_0\to q_0)}\bigotimes_{e_0}|+\rangle_{e_0} = \bigotimes_{e_0}|+\rangle_{e_0}$, the product state must be stabilized by $X_{(f_0\to q_0)}$. Applying such X strings to all the plaquettes, we find that $\bigotimes_{e_0}|+\rangle_{e_0}$ is the $+1$ eigenstate of $X_{(f_0\to q_0)}$ for all $q_0 \neq f_0$, which leads to Eq. (S16). With the above considerations, we find that the imperfect measurement operator $M_{\{s_{p_0}\}}$ only acts on the stabilizer bits ($|B_{p_0} = \pm\rangle$ and $|A_{v_0} = \pm\rangle$) and logical operators only act on the logical bits ($|X_{l_1^*} = \pm\rangle$ and $|X_{l_2^*} = \pm\rangle$). Consequently, any state $|\widetilde{\Psi}\rangle$ in the code space $\widetilde{\mathcal{C}}$ can be expanded under the stabilizer basis to achieve the form:

$$|\widetilde{\Psi}\rangle \propto \left[\exp\left(\frac{\beta}{2}\prod\nolimits'_{p_0} B_{p_0}\right) \bigotimes\nolimits'_{p_0} \sum_{b_{p_0}=\pm} \exp\left(\frac{\beta}{2} b_{p_0}\right) |B_{p_0} = b_{p_0}\rangle\right] \left[\bigotimes\nolimits'_{v_0} |A_{v_0} = +\rangle\right] \bigotimes |L\rangle\,, \tag{S17}$$

where $|L\rangle$ represents the logical qubits and is exactly where the logical information is stored.

SII. DERIVATION OF STATISTICAL MECHANICAL MAPPING

Recall that we have considered a multi-round error correction protocol [5] under imperfect syndrome measurement, listed as follows:

1. Start with an arbitrary state $|\widetilde{\Psi}\rangle$ in $\widetilde{\mathcal{C}}(\beta_0)$.

2. Probabilistic Pauli X error acts at each integer valued time $t = 1, 2, \cdots, T$. The X error at each physical qubit on each time slice occurs independently with probability q. Denote the error configuration at time t as $\{\eta_{e_0}(t)\}$.

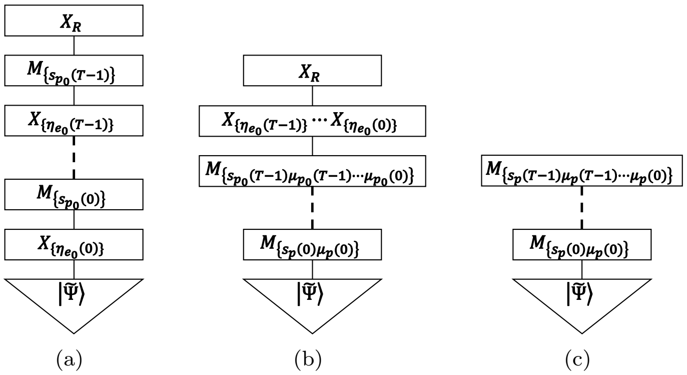

FIG. S3. Diagrams for error correction procedure. The bottom triangle is the initial state. The squares above represent actions of Pauli errors and imperfect measurements. (a) Original error correction procedure. Pauli errors and imperfect measurements act repetitiously. (b) Move Pauli error operators to the top making use of the commutation relation, i.e. Eq. (S18). (c) When the correction operator and Pauli error operators form a stabilizer, they together commute with imperfect measurement operators and hence have no effect on the final state.

Note that $\eta_{e_0}(t) = -1$ marks the presence of Pauli error on the edge e_0 at time t and $\eta_{e_0}(t) = +1$ otherwise. The associated Pauli operator is $X_{\{\eta_{e_0}(t)\}} = \prod_{e_0}(\delta_{\eta_{e_0}(t),+1} I + \delta_{\eta_{e_0}(t),-1} X_{e_0})$.

3. Perform a round of syndrome measurement between each time interval $[t, t+1]$. The syndrome measurements are also imperfect and their strength of measurement is set as β. Suppose the measurement outcome at time interval $[t, t+1]$ is $\{s_{p_0}(t)\}$, then the associated action of imperfect measurement is $M_{\{s_{p_0}(t)\}}$.

4. After T rounds of syndrome measurements, we decode and apply Pauli X correction to the final state. We denote the correction operator as X_R.

Notice that with our definition of code space $\tilde{\mathcal{C}}$, the imperfection of syndrome measurement only affects syndrome bits but does not disturb logical information. We have known that any state in $\tilde{\mathcal{C}}$ takes the form as shown in Eq. (S17). Acting successive imperfect measurement operators on it still only affects the stabilizer bits. The logical information $|L\rangle$ will be left unchanged.

We might represent our error correction procedure as a diagram in Fig. 3(a). We will show that when the error chains of the whole history and the correction chain together constitute a contractible loop (correction operator and Pauli error operators forms stabilize) the logical information is still preserved. Notice that the commutation between $M_{\{s_{p_0}(t)\}}$ and Pauli error $X_{\{\eta_{e_0}(t')\}}$ can be expressed as

$$M_{\{s_{p_0}(t)\}} X_{\{\eta_{e_0}(t')\}} = X_{\{\eta_{e_0}(t')\}} M_{\{s_{p_0}(t)\mu_{p_0}(t')\}}. \tag{S18}$$

Here $\{\mu_{p_0}(t')\}$ represents the correct syndrome that should be generated by Pauli error $X_{\{\eta_{e_0}(t)\}}$. In other words $\mu_{p_0}(t') = -1$ when p locates at the boundary of the error chain $\{\eta_{e_0}(t')\}$ and $\mu_{p_0}(t') = 1$ otherwise. The boundary configuration can also be represented by $\eta_{e_0}(t')$, that is $\mu_{p_0}(t') = \prod_{e_0 \in \partial p_0} \eta_{e_0}(t')$. Using this commutation relation we can move all Pauli error chains in Fig. 3(a) to the top and arrive at Fig. 3(b). When the operator $X_R X_{\{\eta_{e_0}(t)\}} \cdots X_{\{\eta_{e_0}(1)\}}$ forms a product of A_{v_0} operators, it will be commuting with imperfect measurement operators and logical Pauli operators, and hence acts trivially on the state below. Therefore we will obtain a final state as in Fig. 3(c). Though the final state is different from the original state $|\widetilde{\Psi}\rangle$, it will be containing the same logical information, so we view this situation as a success of error correction.

Then we may compute the probability of syndrome outcomes given a particular error configuration $\{\eta_{e_0}(t)\}_t$

$$\begin{aligned} P(\{s_{p_0}(t)\}_t | \{\eta_{e_0}(t)\}_t) &= || \prod_t M_{\{s_{p_0}(t)\}} X_{\{\eta_{e_0}(t)\}} |\widetilde{\Psi}\rangle ||^2 \\ &= || \prod_t M_{\{s_{p_0}(t) \prod_{k \le t} \mu_{p_0}(k)\}} |\widetilde{\Psi}\rangle ||^2 = \langle \tilde{\Psi} | \prod_t E_{\{s_{p_0}(t) \prod_{k \le t} \mu_{p_0}(k)\}} |\widetilde{\Psi}\rangle . \end{aligned} \tag{S19}$$

Here $|| \cdot ||^2$ denotes the state norm and the subscript t in $\{s_{p_0}(t)\}_t$ and $\{\eta_{e_0}(t)\}_t$ remind us that they are the syndrome and Pauli error configurations of all the time steps. First, note that this expression is a well-defined joint probability for the $s_{p_0}(t)$ variables. $P(\{s_{p_0}(t)\}_t | \{\eta_{e_0}(t)\}_t)$ is always non-negative, and it is normalized as

$\sum_{\{s_{p_0}(t)\}_t} P(\{s_{p_0}(t)\}_t|\{\eta_{e_0}(t)\}_t) = 1$, which could be verified by applying the normalization of POVM operators $\sum_{\{s_{p_0}\}} E_{\{s_{p_0}\}} = I$ for each time slice. Besides, we can check that it is the true probability of syndrome measurements on the physical level. For example, imagine we are performing error correction in the real world. At the time t', we ask what the syndrome measurement probability for the current step is. Eq. (S19) tells us that it should be a probability at the t' step conditioned on the configurations of previous steps

$$\begin{aligned} P(\{s_{p_0}(t')\}|\{s_{p_0}(t)\}_{t<t'}, \{\eta_{e_0}(t)\}_t) &= \frac{\sum_{\{s_{p_0}(t)\}_{t>t'}} P(\{s_{p_0}(t)\}_t|\{\eta_{e_0}(t)\}_t)}{\sum_{\{s_{p_0}(t)\}_{t\geq t'}} P(\{s_{p_0}(t)\}_t|\{\eta_{e_0}(t)\}_t)} = \frac{\|\prod_{t\leq t'} M_{\{s_{p_0}(t)\}} X_{\{\eta_{e_0}(t)\}} |\widetilde{\Psi}\rangle \|^2}{\|\prod_{t<t'} M_{\{s_{p_0}(t)\}} X_{\{\eta_{e_0}(t)\}} |\widetilde{\Psi}\rangle \|^2} \\ &= \frac{\mathrm{tr}(E_{\{s_{p_0}(t')\}}\rho)}{\mathrm{tr}(\rho)}, \quad \rho = \left(X_{\{\eta_{e_0}(t')\}} \prod_{t<t'} M_{\{s_{p_0}(t)\}} X_{\{\eta_{e_0}(t)\}} \right) |\widetilde{\Psi}\rangle \langle \widetilde{\Psi}| \left(X_{\{\eta_{e_0}(t')\}} \prod_{t<t'} M_{\{s_{p_0}(t)\}} X_{\{\eta_{e_0}(t)\}} \right)^\dagger . \end{aligned} \tag{S20}$$

We arrive at the actual POVM probability at the current error correction step. Note that it also does not depend on the Pauli errors after time t'. Calculate the expression in Eq. (S19) explicitly, we have

$$\begin{aligned} P(\{s_{p_0}(t)\}_t|\{\eta_{e_0}(t)\}_t) &= \frac{1}{\mathcal{Z}_{\{+\}}} \sum_{\{\sigma_{e_0}\}} \mathrm{e}^{\beta_0 \sum_{p_0} b_{p_0}} \prod_{p_0,t} \frac{\exp\left(\beta b_{p_0} s_{p_0}(t) \prod_{k\leq t} \mu_{p_0}(k)\right)}{2\cosh\beta} \\ &= \frac{1}{\mathcal{Z}_{\{+\}}(2\cosh\beta)^{NT}} \sum_{\{\sigma_{e_0}\}} \exp\left[\sum_{p_0} b_{p_0} \left(\beta_0 + \beta \sum_t s_{p_0}(t) \prod_{k\leq t} \mu_{p_0}(k)\right)\right]. \end{aligned} \tag{S21}$$

Here $\mathcal{Z}_{\{+\}} = 4^N(\cosh^N\beta_0 + \sinh^N\beta_0)$ is the partition function of 2-d $\mathbb{Z}_2$ lattice gauge theory. The σ_{e_0}'s have the same origin as Eq. (S2) through expansion under computational basis. Note that the above expression is independent of the choice of $|\widetilde{\Psi}\rangle$ in code space. This can be shown by expanding $|\widetilde{\Psi}\rangle$ under logical basis $|\widetilde{\Psi}\rangle = \Psi_{++}|\widetilde{++}\rangle + \Psi_{+-}|\widetilde{+-}\rangle + \Psi_{-+}|\widetilde{-+}\rangle + \Psi_{--}|\widetilde{--}\rangle$ and using Elitzur's theorem. Since the POVM operators $E_{s_{p_0}(t)}$ at different times and different plaquettes are all complete and commute with each other, we may view the above probability as a joint probability of syndrome outcomes at different spacetime points, and it is conditioned on the error configuration. Notice that the syndromes at different times are correlated by the physical spin σ_{e_0}. That is because the imperfect measurement at each time step alters the instant quantum state, which affects the syndrome probability at the next time step. We can also construct a joint probability for both syndrome outcomes and error configuration. Noticing that the probability of a given error configuration is

$$P(\{\eta_{e_0}(t)\}_t) = \prod_{e_0,t} q^{\frac{1+\eta_{e_0}(t)}{2}}(1-q)^{\frac{1-\eta_{e_0}(t)}{2}} = \prod_{e_0,t} \frac{\exp(K\eta_{e_0}(t))}{2\cosh K}, \tag{S22}$$

we have

$$\begin{aligned} &P(\{s_{p_0}(t)\}_t, \{\eta_{e_0}(t)\}_t) = P(\{s_{p_0}(t)\}_t|\{\eta_{e_0}(t)\}_t) P(\{\eta_{e_0}(t)\}_t) \\ &= \frac{1}{\mathcal{Z}_{\{+\}}} \prod_{e_0,t} \frac{\exp(K\eta_{e_0}(t))}{2\cosh K} \sum_{\{\sigma_{e_0}\}} \mathrm{e}^{\beta_0 \sum_{p_0} b_{p_0}} \prod_{p,t} \frac{\exp\left(\beta b_{p_0} s_{p_0}(t) \prod_{k\leq t} \prod_{e_0\in\partial p_0} \eta_{e_0}(k)\right)}{2\cosh\beta} \\ &= \frac{1}{\mathcal{Z}_{\{+\}}(2\cosh\beta)^{NT}(2\cosh K)^{2NT}} \sum_{\{\sigma_{e_0}\}} \exp\left[K\sum_{e_0,t}\eta_{e_0}(t) + \sum_{p_0} b_{p_0}\left(\beta_0 + \beta\sum_t s_{p_0}(t) \prod_{k\leq t} \prod_{e_0\in\partial p_0} \eta_{e_0}(k)\right)\right]. \end{aligned} \tag{S23}$$

Here $K = -\frac{1}{2}\ln\frac{q}{1-q}$.

Then we shall discuss in detail how could we decode with the syndrome outcomes $\{s_{p_0}(t)\}_t$. We mainly follow the method used in Ref. [5]. It is convenient to consider the situation that our logical information is stored forever. We extend the initial time $t = 1$ to $-\infty$ and the final time $t = T$ to $+\infty$, which means that the syndrome measurement procedure is performed forever without beginning or end. The error correction procedure is represented as a 3 dimensional lattice in Fig. S4. The vertical plaquettes represent physical qubits at different times and the horizontal plaquettes mark syndrome outcomes. The Pauli X errors are associated with vertical plaquettes (horizontal dashed lines), and the measurement errors are associated with horizontal plaquettes (vertical dashed lines). A given syndrome forms a chain in 3-d spacetime, and we denote the syndrome chain as c_S^*. Note that c_S^* is a set of spacetime plaquettes,

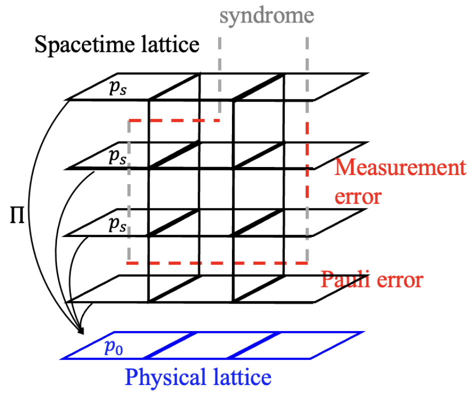

FIG. S4. 3-d spacetime of error history. The black lattice is the spacetime lattice, which takes periodic boundary condition at space directions and infinite boundary condition at time direction. The Pauli errors, measurement errors and error syndrome are represented as strings on the dual lattice (dashed lines). The horizontal red strings crossing timelike (vertical) plaquettes represent the locations of Pauli X errors which actually act on the physical lattice (blue). The vertical gray strings crossing spacelike (horizontal) plaquettes represent the error syndrome, which marks the locations of measurement results $s_{p_0}(t) = -1$ at different time steps. The vertical red strings are where error syndromes do not match the Pauli errors that actually occurred, which we view as measurement errors. Π denotes the projection from the 3-d spacetime lattice to the 2-d physical lattice.

and we view it as a chain on the dual lattice (dashed lines in Fig. S4). We can also view it as a $\mathbb{Z}_2$ valued vector with all the plaquettes as its basis. The task of the decoder is to find out both measurement errors and Pauli errors with respect to the information of syndrome c_S^*. Denote the error chain of both measurement and Pauli errors as c_E^*, it obvious that c_E^* should have the same boundary as c_S^*, $\partial^* c_S^* = \partial^* c_E^*$. Here $\partial^* c_S^*$ is the set of cubics where the boundary points of c_S^* lie in. Suppose the error chain decided by the decoder is $c_{E'}^*$, when $c_{E'}^*$ and c_E^* are homologically equivalent, which means $c_{E'}^* + c_E^*$ forms contractible loop (here the plus is defined mod $\mathbb{Z}_2$), it should make no difference when we finally apply correction operator at $t = +\infty$, hence the error correction will be successful. But if $c_{E'}^* + c_E^*$ forms non-contractible loops, then the corresponding correction operator will be containing logical X operator which has a nontrivial influence on logical information. In that case, the error correction will fail. So the task of the optimal decoder, named as maximum likelihood decoder, is to identify the equivalent class of error chains with the largest probability. Here the equivalent class of an error chain $[c_E^*]$ is defined as the set of all error chains that are homologically equivalent to c_E^*.

The probability of error chain class could be derived from Eq. (S23). Notice that measurement error configuration can be inferred from the syndrome at that moment and error configuration in the past

$$\eta_{p_0}(t) = s_{p_0}(t) \prod_{k \le t} \mu_{p_0}(k) = s_{p_0}(t) \prod_{k \le t} \prod_{e_0 \in \partial p_0} \eta_{e_0}(k). \tag{S24}$$

Substitute the above equation into Eq. (S23), we obtain the joint probability of both measurement and Pauli error

$$\begin{aligned} P(\{\eta_{p_0}(t)\}_t, \{\eta_{e_0}(t)\}_t) &= \frac{1}{\mathcal{Z}_{\{+\}} \prod_t [(2\cosh\beta)^N (2\cosh K)^{2N}]} \\ &\times \sum_{\{\sigma_{e_0}\}} \exp\left[K \sum_{e,t} \eta_{e_0}(t) + \sum_{p_0} b_{p_0}\left(\beta_0 + \beta \sum_t \eta_{p_0}(t)\right)\right]. \end{aligned} \tag{S25}$$

In this expression, we see that measurement errors at different time steps are correlated. Generally, the presence of measurement error increases the probability of measurement error at later times. We reinterpret the above equation on the 3-d lattice. Given an error chain in 3-d spacetime c_E^*, we still mark the location of error as $\eta_p = -1$. Specifically, measurement error at a spacelike plaquette is denoted as $\eta_{p_s} = -1$. Pauli error at a timelike plaquette is denoted as

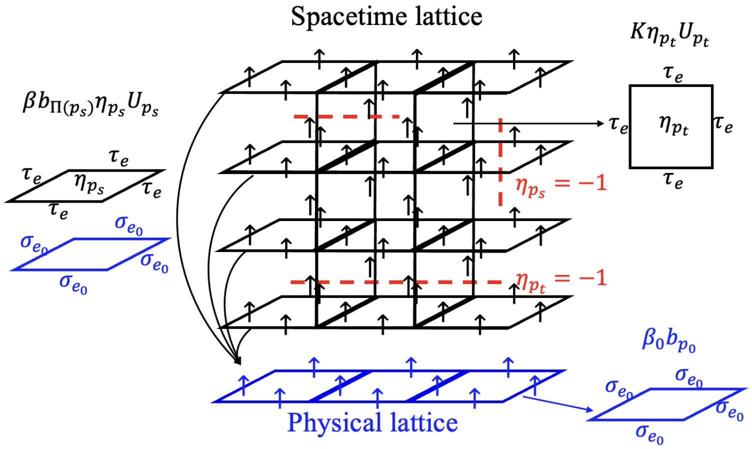

FIG. S5. Illustration of the SM model we obtained. The τ_e spins are defined on the edges of 3-d spacetime lattice and the σ_{e_0}. There are three types of interactions in this model. $\beta_0 b_{p_0}$ is the gauge interaction term defined on the physical lattice. $K\eta_{p_t}U_{p_t}$ is the timelike gauge interaction on spacetime lattice. The $\beta b_{\Pi(p_s)}\eta_{p_s}U_{p_s}$ term couples the spacelike gauge interaction term U_{p_s} to the gauge interaction term $b_{\Pi(p_s)}$ on physical lattice. The η_p's set the signs of gauge interactions on the spacetime lattice.

$\eta_{p_t} = -1$. Then the probability of the error chain is expressed as

$$P(c_E^*) = P(\{\eta_p\}) = \frac{1}{\mathcal{Z}_{\{+\}}\prod_t[(2\cosh\beta)^N(2\cosh K)^{2N}]} \times \sum_{\{\sigma_{e_0}\}} \exp\left[K\sum_{p_t}\eta_{p_t} + \beta_0\sum_{p_0} b_{p_0} + \beta\sum_{p_s} b_{\Pi(p_s)}\eta_{p_s}\right]. \tag{S26}$$

Note that σ_{e_0}'s and associated b_{p_0}'s are viewed as lying on a 2-d lattice distinct from the 3-d spacetime representing error correction history. Each p_s is associated with an $b_{\Pi(p_s)}$ on the 2-d physical lattice such that p_s and $\Pi(p_s)$ correspond to the same space point as in Fig. S4.

The probability of error class $[c_E]$ is calculated as a summation of probabilities of error chains that belong to the same equivalent class:

$$P([c_E]) = \sum_{c\in[c_E]} P(c) \tag{S27}$$

This summation can be done by introducing virtual spin $\tau_p = \pm 1$ associated with each spacetime plaquette and relating it to each edge through $\mathbb{Z}_2$ gauge interaction [5], as in Fig. S5. The summation of homologically equivalent error chains yields the same result as the summation of configurations of virtual spins τ_e defined on edges up to a factor counting the number of 1-form symmetry operations since the sign changing of τ_e corresponds to a deformation of error chain (see Fig. S6) and thus relates homologically equivalent error chains.:

$$\begin{aligned} P([c_E]) &= \frac{1}{\mathcal{N}_1}\sum_{\{\tau_p\}} P(\{\eta_p \prod_{e\in\partial p}\tau_e\}) \\ &= \frac{1}{\mathcal{N}_1\mathcal{Z}_{\{+\}}\prod_t[(2\cosh\beta)^N(2\cosh K)^{2N}]} \\ &\times \sum_{\{\sigma_{e_0}\},\{\tau_e\}} \exp\left[\beta_0\sum_{p_0} b_{p_0} + \beta\sum_{p_s}\left(b_{\Pi(p_s)}\eta_{p_s}\prod_{e\in\partial p_s}\tau_e\right) + K\sum_{p_t}\left(\eta_{p_t}\prod_{e\in\partial p_t}\tau_e\right)\right]. \end{aligned} \tag{S28}$$

Here $\mathcal{N}_1$ denotes the number of 1-form symmetry operations and it diverges for infinite time T. We now arrive at an SM model including both virtual spins $\{\tau_p\}$ on the 3-d spacetime lattice and physical spins $\{\sigma_{e_0}\}$ on a distinct 2-d space lattice. The virtual spins in this SM model describe the fluctuation of error chains in the same class The physical spins are coupled to virtual spins on time-like plaquettes at all times since the probability of measurement errors at

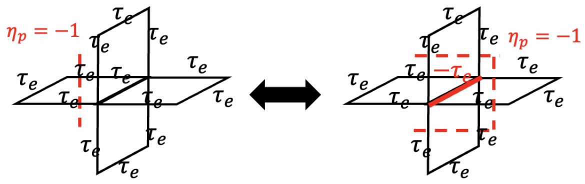

FIG. S6. Example of local symmetry. The red dashed lines represent the position where $\eta_p = -1$. The two $\{\eta_p\}$ configurations yield the same partition function by redefining the spin colored red. Pictorially, flipping τ_e spins is equivalent to a deformation of error chain.

a certain time depends on the entire syndrome measurement history. The optimal decoding algorithm should select the error class $[c_E]$ with the largest $P([c_E])$. Moreover, if the interaction configuration $\{\eta_e\}$ is viewed as disordered with a correlated probability distribution $P(\{\eta_e\})$ defined in equation S26, then the deconfinement-confinement phase transition point of virtual spins $\{\tau_p\}$ in this disordered SM model should signify the error threshold of the error correction protocol as discussed in Ref. [5]. In summary, the quenched disordered SM model describing the error threshold is

$$\begin{aligned} \mathcal{Z}(\{\eta_p\}) &= \sum_{\{\sigma_{e_0}\},\{\tau_e\}} \exp\left[\beta_0 \sum_{p_0} b_{p_0} + \beta \sum_{p_s} b_{\Pi(p_s)} \eta_{p_s} U_{p_s} + K \sum_{p_t} \eta_{p_t} U_{p_t}\right], \\ U_p &= \prod_{e\in\partial p} \tau_e, \quad b_{p_0} = \prod_{e_0 \in \partial p_0} \sigma_{e_0}, \\ P(\{\eta_e\}) &= \frac{\sum_{\{\sigma_{e_0}\}} \exp\left[\beta_0 \sum_{p_0} b_{p_0} + \beta \sum_{p_s} b_{\Pi(p_s)} \eta_{p_s} + K \sum_{p_t} \eta_{p_t}\right]}{\mathcal{Z}_{\{+\}} \prod_t [(2\cosh\beta)^N (2\cosh K)^{2N}]}. \end{aligned} \tag{S29}$$

Note that in our SM model, all the spacelike plaquettes are coupled with the σ_{e_0}'s, causing a highly non-local correlation in the timelike direction. This is a consequence of the imperfect measurement in the initial state preparation. When the initial state is well prepared $\beta_0 \to +\infty$, all the b_{p_0}'s will be set to $+1$ in Eq. (S29) and we arrive at a random plaquette gauge model:

$$\begin{aligned} \mathcal{Z}(\{\eta_p\}) &= \sum_{\{\sigma_{e_0}\},\{\tau_e\}} \exp\left[\beta \sum_{p_s} \eta_{p_s} U_{p_s} + K \sum_{p_t} \eta_{p_t} U_{p_t}\right], \\ U_p &= \prod_{e\in\partial p} \tau_e, \\ P(\{\eta_e\}) &= \frac{\exp\left[K \sum_{p_t} \eta_{p_t} + \beta \sum_{p_s} \eta_{p_s}\right]}{\prod_t [(2\cosh\beta)^N (2\cosh K)^{2N}]}, \end{aligned} \tag{S30}$$

which is derived in Ref. [5] to describe the error threshold under probabilistic measurement error.

From Eq. (S29) we can sum the σ_e's out and arrive at the SM model containing pure τ_e spins

$$\begin{aligned} \mathcal{Z}(\{\eta_p\}) &= \sum_{\{\tau_e\}} \left[\prod_{p_0} \cosh(\beta_0 + \beta \sum_t \eta_{p_s} U_{p_s}) + \prod_{p_0} \sin(\beta_0 + \beta \sum_t \eta_{p_s} U_{p_s})\right] \exp\left[K \sum_{p_t} \eta_{p_t}\right], \\ P(\{\eta_e\}) &= \frac{1}{[(\cosh\beta_0)^N + (\sinh\beta_0)^N] \prod_t [(2\cosh\beta)^N (2\cosh K)^{2N}]} \\ &\times \left[\prod_{p_0} \cosh(\beta_0 + \beta \sum_t \eta_{p_s}) + \prod_{p_0} \sin(\beta_0 + \beta \sum_t \eta_{p_s})\right] \exp\left[K \sum_{p_t} \eta_{p_t}\right]. \end{aligned} \tag{S31}$$

SIII. LOCAL SYMMETRY ON NISHIMORI LINE

The SM model $\mathcal{Z}(\{\eta_p\})$ has a local symmetry when redefining the interaction background $\{\eta_p\}$

$$\eta_p \to \eta_p \prod_{e\in\partial p} \nu_e, \quad \tau_e \to \tau_e \nu_e, \quad \nu_e = \pm 1. \tag{S32}$$

as in Fig. S6. More precisely, the partition function is invariant under redefinition of background interaction configuration $\{\eta_p\}$:

$$\mathcal{Z}(\{\eta_p \prod_{e\in\partial p} \nu_e\}) = \mathcal{Z}(\{\eta_p\}) \tag{S33}$$

since the influence is absorbed in the summation of spin configuration by redefining spin variable $\tau'_e = \tau_e\nu_e$. Note that $\{\eta_p\}$ correspond to a representative error chain in error class $[c_E]$, and the local invariance Eq. (S32) tells us that $\mathcal{Z}(\{\eta_p\})$ does not depend on the choice of representative error chain. It only depends on the homological class of error chain.

We will use the local invariance S32 on Nishimori line to derive several results about the phase structure [6, 7]. Under the presence of disorder, to compute an expectation value, we should first take the ensemble average for a particular interaction configuration (denoted by $\langle\cdot\rangle_{\{\eta_p\}}$) and then take the disorder average on different configurations (denoted as $[\cdot]$). For a given observable $O(\{\eta_p\},\{\sigma_{e_0}\},\{\tau_e\})$ depending on the σ spins, τ spins and interaction configuration, these two kinds of averages are defined as:

$$\begin{aligned} \langle O\rangle_{\{\eta_p\}} &= \frac{1}{\mathcal{Z}(\{\eta_p\})} \sum_{\{\sigma_{e_0}\},\{\tau_e\}} O \exp\left[\beta_0 \sum_{p_0} b_{p_0} + \beta \sum_{p_s} b_{\Pi(p_s)}\eta_{p_s} U_{p_s} + K\sum_{p_t} \eta_{p_t} U_{p_t}\right] \\ [\langle O\rangle_{\{\eta_p\}}] &= \sum_{\{\eta_p\}} P(\{\eta_p\}) \langle O\rangle_{\{\eta_p\}} \end{aligned} \tag{S34}$$

If an observable $O(\{\eta_p\},\{\sigma_{e_0}\},\{\tau_e\})$ is also invariant under local symmetry transformation in Eq. (S32):

$$O(\{\eta_p \prod_{e\in\partial p}\nu_e\},\{\sigma_{e_0}\},\{\tau_e\nu_e\}) = O(\{\eta_p\},\{\sigma_{e_0}\},\{\tau_e\}) \tag{S35}$$

then the expectation value can be evaluated as:

$$\begin{aligned} [\langle O\rangle] &= \sum_{\{\eta_p\}} \frac{P(\{\eta_p\})}{\mathcal{Z}(\{\eta_p\})} \sum_{\{\sigma_{e_0}\},\{\tau_e\}} O(\{\eta_p\},\{\sigma_{e_0}\},\{\tau_e\}) \\ &\times \exp\left[\beta_0 \sum_{p_0} b_{p_0} + \beta \sum_{p_s} b_{\Pi(p_s)}\eta_{p_s} U_{p_s} + K\sum_{p_t} \eta_{p_t} U_{p_t}\right] \\ &= \sum_{\{\eta_p\}} \frac{P(\{\eta_p \prod_{e\in\partial p}\nu_e\})}{\mathcal{Z}(\{\eta_p \prod_{e\in\partial p}\nu_e\})} \sum_{\{\sigma_{e_0}\},\{\tau_e\}} O(\{\eta_p \prod_{e\in\partial p}\nu_e\},\{\sigma_{e_0}\},\{\tau_e\}) \\ &\times \exp\left[\beta_0 \sum_{p_0} b_{p_0} + \beta \sum_{p_s} b_{\Pi(p_s)}\eta_{p_s} U_{p_s} \prod_{e\in\partial p_s}\nu_e + K\sum_{p_t} \eta_{p_t} U_{p_t} \prod_{e\in\partial p_t}\nu_e\right] \\ &= \sum_{\{\eta_p\}} \frac{P(\{\eta_p \prod_{e\in\partial p}\nu_e\})}{\mathcal{Z}(\{\eta_p\})} \sum_{\{\sigma_{e_0}\},\{\tau_e\}} O(\{\eta_p\},\{\sigma_{e_0}\},\{\tau_e\}) \\ &\times \exp\left[\beta_0 \sum_{p_0} b_{p_0} + \beta \sum_{p_s} b_{\Pi(p_s)}\eta_{p_s} U_{p_s} + K\sum_{p_t} \eta_{p_t} U_{p_t}\right] \end{aligned} \tag{S36}$$

$$
\begin{aligned}
&= \frac{1}{2^{3NT}} \sum_{\{\nu_e\}} \sum_{\{\eta_p\}} \frac{P(\{\eta_p \prod_{e\in\partial p} \nu_e\})}{\mathcal{Z}(\{\eta_p\})} \sum_{\{\sigma_{e_0}\},\{\tau_e\}} O(\{\eta_p\},\{\sigma_{e_0}\},\{\tau_e\}) \\
&\times \exp\left[\beta_0 \sum_{p_0} b_{p_0} + \beta \sum_{p_s} b_{\Pi(p_s)} \eta_{p_s} U_{p_s} + K \sum_{p_t} \eta_{p_t} U_{p_t}\right] \\
&= \frac{1}{2^{3NT} \mathcal{Z}_{\{+\}} (2\cosh\beta)^{NT} (2\cosh K)^{2NT}} \sum_{\{\sigma_{e_0}\},\{\tau_e\},\{\eta_p\}} O(\{\eta_p\},\{\sigma_{e_0}\},\{\tau_e\}) \\
&\times \exp\left[\beta_0 \sum_{p_0} b_{p_0} + \beta \sum_{p_s} b_{\Pi(p_s)} \eta_{p_s} U_{p_s} + K \sum_{p_t} \eta_{p_t} U_{p_t}\right]
\end{aligned}
$$

In the third equality, we make use of the local symmetry. In the fourth equality we averaged over different $\{\nu_e\}$ since the final result $[\langle O\rangle]$ is independent of $\{\nu_e\}$. The fifth equality is obtained by noticing that:

$$
\sum_{\{\nu_e\}} P(\{\eta_p \prod_{e\in\partial p} \nu_e\}) = \frac{\mathcal{Z}(\{\eta_p\})}{\mathcal{Z}_{\{+\}} (2\cosh\beta)^{NT} (2\cosh K)^{2NT}} \tag{S37}
$$

First we consider $O = W_{A_0} = \prod_{p_0\in A_0} b_{p_0}$ which is the Wilson loop on 2-d physical lattice. Clearly, it satisfies Eq. (S35). So we calculate its expectation value as:

$$
\begin{aligned}
[\langle W_{A_0}\rangle] &= \frac{1}{2^{3NT} \mathcal{Z}_{\{+\}} (2\cosh\beta)^{NT} (2\cosh K)^{2NT}} \\
&\sum_{\{\sigma_{e_0}\},\{\tau_e\},\{\eta_p\}} W_{A_0} \exp\left[\beta_0 \sum_{p_0} b_{p_0} + \beta \sum_{p_s} b_{\Pi(p_s)} \eta_{p_s} U_{p_s} + K \sum_{p_t} \eta_{p_t} U_{p_t}\right] \\
&= \frac{1}{\mathcal{Z}_{\{+\}}} \sum_{\{\sigma_{e_0}\}} W_{A_0} \exp\left[\beta_0 \sum_{p_0} b_{p_0}\right] = \langle W_{A_0}\rangle_{\{+\}} \\
&= \frac{(\tanh\beta_0)^{|A_0|} + (\tanh\beta_0)^{N-|A_0|}}{1 + (\tanh\beta_0)^N}
\end{aligned} \tag{S38}
$$

Here $\langle\cdot\rangle_{\{+\}}$ denotes the expectation value for the pure 2-d $\mathbb{Z}_2$ gauge theory of physical spins σ_{e_0}, $\mathcal{Z}_{\{+\}} = \sum_{\{\sigma_{e_0}\}} \exp\left[\beta_0 \sum_{p_0} b_{p_0}\right]$. Actually, the above derivation works for any observable contains purely σ_{e_0} spins. So we conclude that the σ_{e_0} spins in SM model shown in Eq. (S29) behaves exactly the same as pure 2-d $\mathbb{Z}_2$ gauge theory. Specifically for the Wilson loop W_{A_0}, we find it obeys area law for any finite β_0, same as 2-d $\mathbb{Z}_2$ lattice gauge theory, so the σ_{e_0} spins always stays in disordered phase.

We may also compute the internal energy, which is also invariant under the transformation shown in Eq. (S32). Given that

$$
\begin{aligned}
[\langle \eta_{p_s} b_{\pi(p_s)} U_{p_s}\rangle] &= \frac{1}{2^{3NT} \mathcal{Z}_{\{+\}} (2\cosh\beta)^{NT} (2\cosh K)^{2NT}} \\
&\times \sum_{\{\sigma_{e_0}\},\{\tau_e\},\{\eta_p\}} \eta_{p_s} b_{\pi(p_s)} U_{p_s} \exp\left[\beta_0 \sum_{p_0} b_{p_0} + \beta \sum_{p_s} b_{\Pi(p_s)} \eta_{p_s} U_{p_s} + K \sum_{p_t} \eta_{p_t} U_{p_t}\right] \\
&= \frac{1}{2^{3NT} \mathcal{Z}_{\{+\}} (2\cosh\beta)^{NT} (2\cosh K)^{2NT}} \sum_{\{\sigma_{e_0}\},\{\tau_e\}} (2\sinh\beta)(2\cosh\beta)^{NT-1} (2\cosh K)^{2NT} \exp\left[\beta_0 \sum_{p_0} b_{p_0}\right] \\
&= \tanh\beta,
\end{aligned} \tag{S39}
$$

and similarly

$$\begin{aligned}
[\langle \eta_{p_t} U_{p_t} \rangle] &= \frac{1}{2^{3NT}\mathcal{Z}_{\{+\}}(2\cosh\beta)^{NT}(2\cosh K)^{2NT}} \\
&\times \sum_{\{\sigma_{e_0}\},\{\tau_e\},\{\eta_p\}} \eta_{p_t} U_{p_t} \exp\left[\beta_0 \sum_{p_0} b_{p_0} + \beta \sum_{p_s} b_{\Pi(p_s)} \eta_{p_s} U_{p_s} + K \sum_{p_t} \eta_{p_t} U_{p_t}\right] \\
&= \frac{1}{2^{3NT}\mathcal{Z}_{\{+\}}(2\cosh\beta)^{NT}(2\cosh K)^{2NT}} \sum_{\{\sigma_{e_0}\},\{\tau_e\}} (2\sinh K)(2\cosh\beta)^{NT}(2\cosh K)^{2NT-1} \exp\left[\beta_0 \sum_{p_0} b_{p_0}\right] \\
&= \tanh K,
\end{aligned} \tag{S40}$$

we have the expression for internal energy

$$\begin{aligned}
\mathcal{U} &= [\langle -\beta_0 \sum_{p_0} b_{p_0} - \beta \sum_{p_s} b_{\Pi(p_s)} \eta_{p_s} U_{p_s} - K \sum_{p_t} \eta_{p_t} U_{p_t} \rangle] \\
&= -N\beta_0 \frac{\tanh\beta_0 + (\tanh\beta_0)^{N-1}}{1 + (\tanh\beta_0)^N} - NT\beta\tanh\beta - 2NTK\tanh K.
\end{aligned} \tag{S41}$$

Then consider the Wilson loop for τ_e spins $W_A = \prod_{p\in A} U_p = \prod_{e\in\partial A} \tau_e$. Note that it is not invariant under Eq. (S32). By a similar calculation as Eq. (S36) we have:

$$\begin{aligned}
[\langle W_A \rangle] &= \sum_{\{\eta_p\}} \frac{P(\{\eta_p\})}{\mathcal{Z}(\{\eta_p\})} \sum_{\{\sigma_{e_0}\},\{\tau_e\}} W_A \exp\left[\beta_0 \sum_{p_0} b_{p_0} + \beta \sum_{p_s} b_{\Pi(p_s)} \eta_{p_s} U_{p_s} + K \sum_{p_t} \eta_{p_t} U_{p_t}\right] \\
&= \sum_{\{\eta_p\}} \frac{P(\{\eta_p \prod_{e\in\partial p} \nu_e\})}{\mathcal{Z}(\{\eta_p\})} \sum_{\{\sigma_{e_0}\},\{\tau_e\}} W_A \prod_{e\in\partial A} \nu_e \\
&\times \exp\left[\beta_0 \sum_{p_0} b_{p_0} + \beta \sum_{p_s} b_{\Pi(p_s)} \eta_{p_s} U_{p_s} + K \sum_{p_t} \eta_{p_t} U_{p_t}\right] \\
&= \frac{1}{2^{3NT}} \sum_{\{\nu_e\}} \sum_{\{\eta_p\}} \frac{P(\{\eta_p \prod_{e\in\partial p} \nu_e\}) \prod_{e\in\partial A} \nu_e}{\mathcal{Z}(\{\eta_p\})} \sum_{\{\sigma_{e_0}\},\{\tau_e\}} W_A \\
&\times \exp\left[\beta_0 \sum_{p_0} b_{p_0} + \beta \sum_{p_s} b_{\Pi(p_s)} \eta_{p_s} U_{p_s} + K \sum_{p_t} \eta_{p_t} U_{p_t}\right] \\
&= \frac{1}{2^{3NT}\mathcal{Z}_{\{+\}}(2\cosh\beta)^{NT}(2\cosh K)^{2NT}} \sum_{\{\eta_p\}} \mathcal{Z}(\{\eta_p\}) \langle W_A \rangle^2 \\
&= [\langle W_A \rangle^2]
\end{aligned} \tag{S42}$$

The result $[\langle W_A \rangle] = [\langle W_A \rangle^2]$ signifies the absence of gauge glass phase ($[\langle W_A \rangle] = 0$, $[\langle W_A \rangle^2] > 0$) on the Nishimori line [7].

In addition, one may find that the modified Wilson loop $W_A \prod_{p\in A} \eta_p$ is invariant under local symmetry. It can be

calculated as

$$
\begin{aligned}
&[\langle W_A \prod_{p\in A}\eta_p\rangle] = \frac{1}{2^{3NT}\mathcal{Z}_{\{+\}}(2\cosh\beta)^{NT}(2\cosh K)^{2NT}} \\
&\times \sum_{\{\sigma_{e_0}\},\{\tau_e\},\{\eta_p\}} W_A \prod_{p\in A}\eta_p \exp\left[\beta_0\sum_{p_0} b_{p_0} + \beta\sum_{p_s} b_{\Pi(p_s)}\eta_{p_s}U_{p_s} + K\sum_{p_t}\eta_{p_t}U_{p_t}\right] \\
&= \frac{1}{2^{3NT}\mathcal{Z}_{\{+\}}(2\cosh\beta)^{NT}(2\cosh K)^{2NT}} \sum_{\{\sigma_{e_0}\},\{\tau_e\}} \exp\left[\beta_0\sum_{p_0} b_{p_0}\right] \\
&\times \left(\prod_{p_s\in A_s}\sum_{\eta_{p_s}}\eta_{p_s}U_{p_s}\exp\left[\beta b_{\Pi(p_s)}\eta_{p_s}U_{p_s}\right]\prod_{p_s\notin A_s}\sum_{\eta_{p_s}}\exp\left[\beta b_{\Pi(p_s)}\eta_{p_s}U_{p_s}\right]\right) \\
&\times \left(\prod_{p_t\in A_t}\sum_{\eta_{p_t}}\eta_{p_t}U_{p_t}\exp\left[K\eta_{p_t}U_{p_t}\right]\prod_{p_t\notin A_t}\sum_{\eta_{p_t}}\exp\left[K\eta_{p_t}U_{p_t}\right]\right) \\
&= \frac{1}{2^{3NT}\mathcal{Z}_{\{+\}}(2\cosh\beta)^{NT}(2\cosh K)^{2NT}} \sum_{\{\sigma_{e_0}\},\{\tau_e\}} \exp\left[\beta_0\sum_{p_0} b_{p_0}\right] \\
&\times \left(\prod_{p_s\in A_s} b_{\pi(p_s)}\right)(2\sinh\beta)^{|A_s|}(2\cosh\beta)^{NT-|A_s|}(2\sinh K)^{|A_t|}(2\cosh K)^{2NT-|A_t|} \\
&\langle W_{\Pi(A)}\rangle_{\{+\}}(\tanh\beta)^{|A_s|}(\tanh K)^{|A_t|}
\end{aligned}
\tag{S43}
$$

Here A_s and A_t denote the sets of spacelike plaquettes and timelike plaquettes contained in A respectively. Π is the projection onto physical lattice, see Fig. S9. This expression reveals that τ_e's and σ_e's are correlated and their Wilson loops somehow depend on each other. However, the modified Wilson loop $W_A\prod_{p\in A}\eta_p$ cannot serve as the correct order parameter for the error threshold.

SIV. LOW TEMPERATURE EXPANSION

The ordered (deconfinement) phase is expected to exist at the exact zero temperature point (on the Nishimori line) $\beta_0, \beta, K \to +\infty$. Near the zero temperature point, we may perform a low-temperature expansion for Wilson loops to show if the ordered phase still persists to some finite temperature. Assume $e^{-\beta_0}$, $e^{-\beta}$, e^{-K} are of the same order, we expand $[\langle W_A\rangle]$ up to the order $e^{-4\beta}$ following the method discussed in Ref. [3].

We first perform the expansion for the disorder probability $P(\{\eta_p\})$. Through the expression of $P(\{\eta_p\})$

$$
P(\{\eta_e\}) = \frac{2^{N+1}\sum_{\{b_{p_0}\}}\frac{1+\prod_{p_0}b_{p_0}}{2}\exp\left[\beta_0\sum_{p_0}b_{p_0} + \beta\sum_{p_s}b_{\Pi(p_s)}\eta_{p_s} + K\sum_{p_t}\eta_{p_t}\right]}{\mathcal{Z}_{\{+\}}\prod_t[(2\cosh\beta)^N(2\cosh K)^{2N}]}. \tag{S44}
$$

We notice that the zeroth order is contributed by the configuration that all b_{p_0} and η_p are equal to $+1$. The order $e^{-2\beta}$ is given by flipping one of the η_ps to -1. The order $e^{-4\beta}$ is given by flipping any two of the η_ps or flipping two of b_{p_0}s. Note that the parity of $\{b_{p_0}\}$ configuration must be even. Then we find that the numerator is expanded as

$$
\begin{aligned}
&2^{N+1}E^{N\beta_0+NT\beta+2NTK}\left[\delta_{\{+\}} + e^{-2K}\sum_{p_t}\delta_{\{p_t\}} + e^{-2\beta}\sum_{p_s}\delta_{\{p_s\}}\right. \\
&\left.+e^{-4K}\sum_{(p_t,p_t')}\delta_{\{(p_t,p_t')\}} + e^{-4\beta}\sum_{(p_s,p_s')}\delta_{\{(p_s,p_s')\}} + e^{-2\beta-2K}\sum_{(p_s,p_t)}\delta_{\{(p_s,p_t)\}} + e^{-4\beta_0}\sum_{(p_0,p_0')}\delta_{\{\Pi^{-1}(p_0,p_0')\}}\right].
\end{aligned}
\tag{S45}
$$

Here (p,p') denotes a pair of different plaquettes. For example (p_s,p_s') denotes a pair of spacelike plaquettes and the summation $\sum_{(p_s,p_s')}$ is taken on all such pairs. We have used a simplified notation for the Kronecker delta

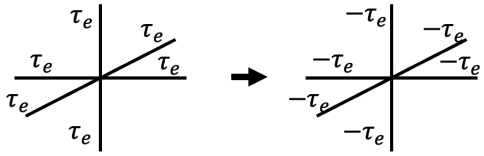

FIG. S7. Example of 1-form symmetry operation. The τ_e spins on a closed surface (surrounding the center vertex in this figure) are flipped.

symbol. δ_+ means that all the η_ps are fixed to be +1, $\delta_+ = \prod_p \delta_{\eta_p,+}$. δ_p denotes that η_p one the given plaquette p is flipped to -1, $\delta_p = \delta_{\eta_p,-} \prod_{p' \neq p} \delta_{\eta_{p'},+}$, and similar for $\delta_{(p,p')}$, $\delta_{(p,p')} = \delta_{\eta_p,-}\delta_{\eta_{p'},-} \prod_{p'' \neq p,p'} \delta_{\eta_{p''},+}$. Finally $\delta_{\Pi^{-1}(p_0,p_0')}$ means that all the spacelike plaquettes whose projection on the physical lattice is p_0 or p_0' are set to be -1, $\delta_{\Pi^{-1}(p_0,p_0')} = \prod_{p_s|\Pi(p_s)=p_0} \delta_{\eta_{p_s},-} \prod_{p_s'|\Pi(p_s')=p_0'} \delta_{\eta_{p_s'},-} \prod_{p_s''|\Pi(p_s'')\neq p_0,p_0'} \delta_{\eta_{p_s},+}$. The number of integer times T should be sent to $+\infty$ at the end of the calculation. Combined with the denominator, we arrive at a perturbative expression for disorder probability

$$
\begin{aligned}
P(\{\eta_p\}) \simeq & \big[1 - \left(1 - 2NTe^{-2K} - NTe^{-2\beta}\right)\left(2NTe^{-2K} + NTe^{-2\beta}\right) \\
& - \left(\binom{2NT}{2} e^{-4K} + \binom{NT}{2} e^{-4\beta} + 2(NT)^2 e^{-2K-2\beta} + \binom{N}{2} e^{-4\beta_0}\right)\bigg] \delta_{\{+\}} \\
& + \left(1 - 2NTe^{-2K} - NTe^{-2\beta}\right)\left[e^{-2K}\sum_{p_t}\delta_{\{p_t\}} + e^{-2\beta}\sum_{p_s}\delta_{\{p_s\}}\right] \\
& + e^{-4K}\sum_{(p_t,p_t')}\delta_{\{(p_t,p_t')\}} + e^{-4\beta}\sum_{(p_s,p_s')}\delta_{\{(p_s,p_s')\}} + e^{-2\beta-2K}\sum_{(p_s,p_t)}\delta_{\{(p_s,p_t)\}} + e^{-4\beta_0}\sum_{(p_0,p_0')}\delta_{\{\Pi^{-1}(p_0,p_0')\}}.
\end{aligned}
\tag{S46}
$$

Then we analyze the ensemble-averaged value for the interaction configurations that appeared in the above expression. Keeping in mind the fact that the interaction term U_p and Wilson loop W_A are both invariant under 1-form symmetry operations which flip the spins on a 2-d surface on the dual lattice as in Fig. S7, when performing the spin configuration summation for the lowest few orders we extract a factor of 1-form symmetry operation numbers $\mathcal{N}_1$ and evaluate the coefficient for each order on an equivalent class of spin configurations module 1-form symmetry.

First, consider the case all $\eta_p = +1$, and we want to compute

$$
\langle W_A \rangle_{\{+\}} = \frac{1}{\mathcal{Z}(\{+\})} \sum_{\{\sigma_{e_0}\},\{\tau_e\}} \left(\prod_{e\in\partial A}\tau_e\right) \exp\left[\beta_0 \sum_{p_0} b_{p_0} + \beta \sum_{p_s} b_{\Pi(p_s)} U_{p_s} + K \sum_{p_t} U_{p_t}\right]. \tag{S47}
$$

The 0th order is contributed by the ground states, and a representative spin configuration is that all $\tau_e = +1$ and $b_{p_0} = +1$. It leads to the term:

$$
\mathcal{N}_1 2^{N+1} e^{N\beta_0 + NT\beta + 2NTK} \tag{S48}
$$

in both the numerator and denominator. Now if we flip one of the τ_e spins to -1, it increases the energy of four neighbor plaquettes and results in a term of order $e^{-8\beta}$, which is not included in our expansion. However, if we flip two b_{p_0}s, this only yields a term of order $e^{-4\beta_0}$ in the infinite boundary condition case. When two b_{p_0}s are flipped, it leads to -1 interactions on two strings of spacelike plaquettes corresponding to the position of b_{p_0}, as shown in S8, and it should cost infinite energy. However, the energy cost can be eliminated by the local symmetry transformation in Eq. (S32). The two strings can be viewed as merged together by setting the τ_e spins on the surface connecting them to -1. Deformation of the surface is nothing but a 1-form symmetry operation and has already been mod out. Combining both the b_{p_0} configuration and the τ_e spin configuration, the total energy cost is just $4\beta_0$. Now consider the value of the Wilson loop. During the process of merging two strings together, if they cross the Wilson loop for odd times, then exactly one of the spins on the boundary of region A will be flipped to -1. So the value of the Wilson loop for that configuration is -1. But if the Wilson loop is crossed for even times, it still gets $+1$. The total contribution of order $e^{-4\beta_0}$ is obtained by summing all the choices for flipping two b_{p_0}s, which contains $\binom{N}{2}$ terms.

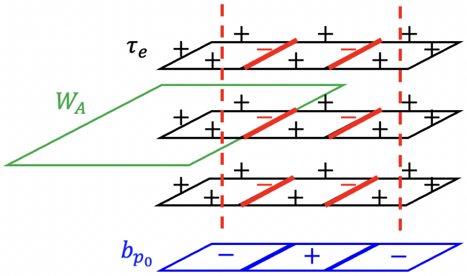

FIG. S8. Spin configuration when two b_{p_0} are flipped. The $\{b_{p_0}\}$ configuration is shown on the blue lattice. The timelike plaquettes are omitted for explicitness. The two red dashed strings represent the locations of -1 plaquette interaction caused by $b_{\Pi(p_s)} = -1$, and their energy cost can be eliminated by flipping the spins on the solid red edges.

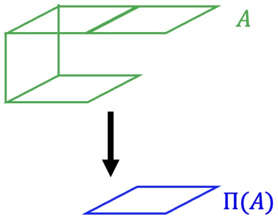

FIG. S9. The action of projection Π on surface A. The above surface lies inside 3-d spacetime and its projection on 2-d space is shown below. Their boundaries correspondingly are Wilson loops in 3-d spacetime and 2-d space.

By counting the number of odd time crosses, we obtain the expression for an additional term in the numerator

$$\left(\binom{N}{2} - 2|\Pi(A)|(N - |\Pi(A)|)\right) e^{-4\beta_0}. \tag{S49}$$

Here we define $\Pi(A)$ as the projection of region A on the spacelike 2-d lattice module $\mathbb{Z}_2$. The timelike plaquettes are dropped under projection, and an even number of spacelike plaquettes on the same space position also leads to zero, as in Fig. S9. For denominator, the new term is just $\binom{N}{2}e^{-4\beta_0}$. So the expansion of $\langle W_A \rangle_{\{+\}}$ is evaluated as

$$\begin{aligned}\langle W_A \rangle_{\{+\}} &\simeq \frac{\mathcal{N}_1 2^{N+1} e^{N\beta_0 + NT\beta + 2NTK}\left[1 + \left(\binom{N}{2} - 2|\Pi(A)|(N - |\Pi(A)|)\right) e^{-4\beta_0}\right]}{\mathcal{N}_1 2^{N+1} e^{N\beta_0 + NT\beta + 2NTK}\left[1 + \binom{N}{2} e^{-4\beta_0}\right]} \\ &\simeq 1 - 2|\Pi(A)|(N - |\Pi(A)|)e^{-4\beta_0}.\end{aligned} \tag{S50}$$

From this expression, we can roughly say that $\langle W_A \rangle_{\{+\}}$ decays with respect to the spacelike area.

Now we evaluated the Wilson loop configurations that only one η_p is flipped. Suppose a timelike plaquette p_t is flipped. Now the ground state energy is $2K$ higher than the previous all $\eta_p = +1$ case and the ground state spin configuration remains the same, which is all $\tau_e = +1$. The lowest excitation is caused by flipping one of the spins at the boundary of p_t, which has the extra energy $4K$. It eventually results in terms of order e^{-4K}. Notice that the probability of $\eta_{p_t} = -1$ is already of the order e^{-2K}, so we only need to concern about the ground state in our expansion, and it leads to

$$\langle W_A \rangle_{\{p_t\}} \simeq 1. \tag{S51}$$

Similarly, for the spacelike plaquette flipping, we also have

$$\langle W_A \rangle_{\{p_s\}} \simeq 1. \tag{S52}$$

Then we consider the configuration where two η_ps are flipped. Notice that only the ground state contribution survives in our expansion since the probability is already of the order $e^{-4\beta}$. We the two flipped η_ps do not contact each other, the representative spin configuration for ground state is still all $\tau_e = +1$ and only those two plaquettes

where $\eta_p = -1$ have energy costs. But when the two flipped plaquettes are nearest neighbors, there will be two degenerate (ignoring the energetic difference between β and K) configurations that both have energy costs on two plaquettes. They are distinguished by whether the contacting edge of the two plaquettes is flipped to -1. If the contacting edges lie on the Wilson loop, then summing the two degenerate configurations leads to $W_A = 0$. So assume the two flipped plaquettes are p and p', then the Wilson loop takes the value

$$\langle W_A \rangle_{\{(p,p')\}} \simeq 1 - |\partial A \cap \partial p \cap \partial p'|. \tag{S53}$$

If the two plaquettes and the Wilson loop intersect at one edge, $|\partial A \cap \partial p \cap \partial p'|$ takes 1. Otherwise, it takes 0. Consider the disorder summation of these configurations. Each edge connects to 4 plaquettes, yielding 6 pairs of nearest-neighbor plaquettes. By noticing that each timelike edge is associated with 6 pairs of timelike plaquettes while each spacelike edge is associated with a pair of timelike plaquettes, a pair of spacelike plaquettes and 4 combinations of spacelike and timelike plaquettes, we find that

$$\begin{aligned} e^{-4\beta} \sum_{(p_s,p_s')} \langle W_A \rangle_{\{(p_s,p_s')\}} &\simeq e^{-4\beta} \left(\binom{NT}{2} - |\partial A|_s \right) \\ e^{-4K} \sum_{(p_t,p_t')} \langle W_A \rangle_{\{(p_t,p_t')\}} &\simeq e^{-4K} \left(\binom{2NT}{2} - |\partial A|_s - 6|\partial A|_t \right) \\ e^{-2\beta-2K} \sum_{(p_t,p_s)} \langle W_A \rangle_{\{(p_t,p_s)\}} &\simeq e^{-2\beta-2K} \left(2(NT)^2 - 4|\partial A|_s \right). \end{aligned} \tag{S54}$$

Here $|\partial A|_s$ denotes the number of spacelike edges in ∂A and $|\partial A|_t$ is the number of timelike edges in ∂A.

Finally we turn to the configuration denoted as $\{\Pi^{-1}(p_0, p_0')\}$, which means all the spacelike interactions η_{p_s} along the timelike strings with the same space position as p_0 or p_0' are flipped. In this case, a similar argument while evaluating Eq. (S50) could be applied. The two timelike strings are merged together by setting the spins in the interval to -1, which gives us the ground state configuration. So the leading order of the Wilson loop is

$$\langle W_A \rangle_{\{\Pi^{-1}(p_0,p_0')\}} \simeq \pm 1. \tag{S55}$$

which takes -1 when only one of the p_0 and p_0' stays in $\Pi(A)$ and takes $+1$ otherwise. Performing the disorder summation, we find that

$$e^{-4\beta_0} \sum_{(p_0,p_0')} \langle W_A \rangle_{\{\Pi^{-1}(p_0,p_0')\}} \simeq e^{-4\beta_0} \left(\binom{N}{2} - 2|\Pi(A)|(N - |\Pi(A)|) \right). \tag{S56}$$

Putting all these stuff together, we arrive at the low-temperature expansion for the Wilson loop

$$\begin{aligned} [\langle W_A \rangle] &\simeq 1 - 4|\Pi(A)|(N - |\Pi(A)|)e^{-4\beta_0} - |\partial A|_s \left(e^{-4\beta} + e^{-4K} + 4e^{-2\beta-2K} \right) - 6|\partial A|_t e^{-4K} \\ &\simeq \exp\left[-4|\Pi(A)|(N - |\Pi(A)|)e^{-4\beta_0} - |\partial A|_s \left(e^{-4\beta} + e^{-4K} + 4e^{-2\beta-2K} \right) - 6|\partial A|_t e^{-4K} \right]. \end{aligned} \tag{S57}$$

Note that the factor $|\Pi(A)|(N - |\Pi(A)|)$ appears because our space manifold is a closed surface. The result is natural to be symmetric between $\Pi(A)$ and the 2-d complement of $\Pi(A)$. We also notice that the number of integer time T automatically vanishes in these expressions so they already suit the case $T \to +\infty$.

From Eq. (S57) we can see that once the initial state preparation suffers from imperfect measurement, the scaling behavior of Wilson loops becomes anisotropic for space and time direction. For a Wilson loop A purely containing spacelike plaquettes, we have

$$[\langle W_A \rangle] \simeq \exp\left[-4|A|(N - |A|)e^{-4\beta_0} - |\partial A| \left(e^{-4\beta} + e^{-4K} + 4e^{-2\beta-2K} \right) \right]. \tag{S58}$$

For large enough N and large enough Wilson loop satisfying $|A| < N/2$, the area law decaying term dominates and the perimeter term can be ignored. So we conclude that the spacelike Wilson loop always decays with respect to the area at finite temperature even when the temperature is sufficiently low. Equivalently we can say that once the initial state is prepared under imperfect measurement, the spacelike Wilson loop always obeys area law. However, for a timelike Wilson loop, we will get:

$$[\langle W_A \rangle] \simeq \exp\left[-\left(e^{-4\beta} + e^{-4K} + 4e^{-2\beta-2K} \right) |\partial A|_s - 6e^{-4K}|\partial A|_t \right]. \tag{S59}$$

so there will be a finite temperature phase for it to exhibit perimeter law scaling.

As we mentioned in the main text, the subtlety of Eq. (S57) is that we cannot directly take the thermodynamic limit $N \to \infty$. However, we may infer the thermodynamic result in analogy to the 2-d $\mathbb{Z}_2$ gauge theory. We may expand the Wilson loop for 2-d $\mathbb{Z}_2$ gauge theory Eq. (S38) also up to $e^{-4\beta_0}$:

$$\langle W_{A_0} \rangle_{\{+\}} \simeq 1 - 2|A_0|(N - |A_0|)e^{-4\beta_0}. \tag{S60}$$

Compared with Eq. (S57) we find an approximate relation under low temperatures:

$$[\langle W_A \rangle] \simeq \langle W_{\Pi(A)} \rangle^2_{\{+\}} \exp\left[-|\partial A|_s \left(e^{-4\beta} + e^{-4K} + 4e^{-2\beta-2K}\right) - 6|\partial A|_t e^{-4K}\right]. \tag{S61}$$

In this expression $[\langle W_A \rangle]$ is a Wilson loop for τ_e spins and it relates to a Wilson loop $\langle W_{\Pi(A)} \rangle_{\{+\}}$ for σ_{e_0} spins in the 2-d physical lattice. Recall that $\langle \cdot \rangle_{\{+\}}$ denotes the expectation value for 2-d $\mathcal{Z}_2$ gauge theory $\mathcal{Z}_{\{+\}} = \sum_{\{\sigma_{e_0}\}} \exp\left(\beta_0 \sum_{p_0} b_{p_0}\right)$. The expression in Eq. (S61) absorbs the size of space N and we expect it to valid under the thermodynamic limit. Turn back to Eq. (S38). If we take the $N \to +\infty$ limit first (keeping A_0 a finite region) and then perform low-temperature expansion, we arrive at

$$\langle W_{A_0} \rangle_{\{+\}} \simeq 1 - 2|A_0|e^{-2\beta_0} + 2|A_0|^2 e^{-4\beta_0}. \tag{S62}$$

The difference between Eq. (S60) and Eq. (S62) arise from the criticality at zero temperature $\beta_0 = +\infty$. The $e^{-2\beta_0}$ term exhibit a discontinuity at zero temperature, which leads to the non-commuting of $N \to +\infty$ limit and $\beta_0 \to +\infty$ limit. A similar critical behavior should be found in $[\langle W_A \rangle]$. If the thermodynamic limit is taken before the low-temperature expansion, we infer that (not a rigorous proof)

$$[\langle W_A \rangle] \simeq \exp\left[-4|\Pi(A)|e^{-2\beta_0} + 4|\Pi(A)|^2 e^{-4\beta_0} - |\partial A|_s \left(e^{-4\beta} + e^{-4K} + 4e^{-2\beta-2K}\right) - 6|\partial A|_t e^{-4K}\right]. \tag{S63}$$

In all, we expect that spacelike Wilson loops still decay with respect to area under the thermodynamic limit. Another way to think about the area law of spacelike Wilson loops when $N \to +\infty$ is to reconsider an open boundary condition at the spacelike directions and take an infinitely large system size before calculating the low-temperature expansion. Then an odd number of flipped b_{p_0} is allowed since we can send one in a pair of b_{p_0}'s to the infinity and eliminate it. Thus up to the leading order we have $[\langle W_A \rangle] \simeq \exp\left[-4|\Pi(A)|e^{-2\beta_0}\right]$. Although this open boundary condition does not correspond to any QEC procedure, we expect that the phase structure of the SM model does not depend on the choice of spacelike boundary conditions, and the expectation values of the order parameter for both the two kinds of boundary conditions should match under the thermodynamic limit.

In addition, Eq. S61 implies that the area law behavior of spacelike Wilson loops is controlled by the finite temperature disordered phase of the 2-d physical σ_{e_0} spins. Note that the area law behavior of the Wilson loop $\langle W_{A_0} \rangle_{\{+\}}$ is used in Ref. [1, 2] to argue the absence of long-range entanglement in the imperfect initial state. So in some sense, Eq. (S61) relates the inability of correcting measurement errors to the absence of long-range entanglement through σ_{e_0} spins.

SV. BEHAVIOR OF THE LOGICAL FIDELITY

As we have mentioned in the main text, the measurement errors are unidentifiable even at low temperatures, in the sense that the multi-round syndrome measurement protocol will not be better than a single-round one, i.e. $T = 1$ or a 2D decoder. So one might ask how the QEC behaves when $T = 1$.

Suppose the QEC initial state is the imperfect logical 00 state $|\widetilde{\Psi}\rangle = |\widetilde{00}\rangle = (|\widetilde{++}\rangle + |\widetilde{+-}\rangle + |\widetilde{-+}\rangle + |\widetilde{--}\rangle)/2$, we estimate the impact of measurement errors on the logical fidelity, that is the fidelity between the final state and the initial state. Consider the scenario where the temperature is low and the system is of considerable size. In accordance with the preceding discourse, A Pauli error on a single physical qubit will be confounded with measurement errors having the same syndrome by the decoder. Consequently, the Pauli error will remain uncorrected. If the uncorrected Pauli error intersects with a logical Pauli Z operator, it acts as a logical error on the logical information $|L\rangle$ (refer to Eq. (S17)), leading to a logical fidelity ~ 0. But if the Pauli error locates elsewhere on the lattice, one may check that the effects of the Pauli error and the measurement operator with the same syndrome complement each other and lead to a fidelity ~ 1, since they both flip the same stabilizer bits in Eq. (S17) but do not affect the logical information $|L\rangle$. Average all error configurations, since the number of configurations that a Pauli error intersects with logical Z is proportional to $d = \sqrt{N}$, we anticipate that the logical fidelity behaves as $1 - const \times d$. The logical fidelity is

suppressed by a large distance, which is a signal that the QEC system is above the true threshold (measurement error threshold in our work). Here the constant depends on the physical error rates β_0, β and K but does not depend on the distance d, and it should drop to 0 when the initial state is ideal, $\beta_0 \to +\infty$.

In contrast, we compare it with the $T = 1$ case of Ref. [5], the 2D decoder suffering from stochastic measurement errors. The decoder in this case also mixes up a Pauli error with probabilistic measurement errors, but their effects do not complement each other, since the probabilistic measurement error is just noise on the classical readouts and does not affect the quantum state. Consequently the logical fidelity scales as $1 - const \times d^2$, where the constant depends on the probability of Pauli and measurement errors. This logical fidelity is also above the threshold. Surprisingly, it is worse than the one of our model. However, the logical fidelity under stochastic errors can be improved by increasing T and arriving at an effective QEC when $T \gg d$ [5], which is not possible in our model.

One might argue that although the non-local measurement errors suppress logical fidelity, the correction of other local errors might lead to other terms that increase with d and compete with non-local measurement errors. However, the non-local measurement error suppression should be the leading order contribution in the low-temperature limit. Moreover, if the system lies above the blue crossover in the light red region in Fig. 5(b) of the main text, the effect of non-local measurement errors overweights other local errors. Thus we believe that the non-local measurement error suppression could overweight other terms in that region. Nonetheless, these statements are not rigorous proofs and require further studies.

SVI. INDEPENDENT AND IDENTICAL DISTRIBUTED (IID) COHERENT DEVIATION

Here we discuss the case where the rotation angle in the imperfect measurement model is IID instead of fixed to a specific value. In experiments, the implementation of multi-qubit gates unavoidably suffers from over or under rotation. In the simplified imperfect measurement model we used (Fig. 1 (c) in the main text), $t = \pi/4 + \delta t$ while δt is the coherent deviation and $\delta t = 0$ corresponds to perfect projective measurement. It is more physical to assume δt as independently Gaussian distributed, with zero mean value and small standard deviation σ. The effective imperfect measurement operator on each plaquette reads [1]

$$M_{s_p} \propto \frac{1}{\sqrt{2}}(\cos t + s_p B_p \sin t), \tag{S64}$$

where $s_p = \pm 1$ is the outcome. Note that all discussion in our main text depends only on the POVM operator,

$$E_{s_p} = M_{s_p}^\dagger M_{s_p} = \frac{1}{2}\left(1 + s_p B_p \cos 2\delta t\right), \tag{S65}$$

since it determines the outcome probability of measurement. Although δt is a random variable with zero mean value, we can see that $\cos 2\delta t$ is an even function, $\cos 2\delta t = \cos(-2\delta t)$, so the effective measurement strength will depend on the variance. As an approximation, we may average it over δt and find that

$$\overline{E_{s_p}} = \frac{1}{2}\left(1 + s_p B_p e^{-2\sigma^2}\right). \tag{S66}$$

It is a projective measurement only when $\sigma = 0$. We may also cast it into the form

$$\overline{E_{s_p}} \propto e^{\beta s_p B_p}, \quad \beta = \operatorname{arctanh} e^{-2\sigma^2}, \tag{S67}$$

and it will lead to similar results as in the main text. Although more strictly speaking, we should treat δt as a quenched disorder in the SM model, it will lead to a much harder calculation and we believe that it will not lead to a qualitative difference.

SVII. CODE STATE PREPARATION PROCEDURE TO STAY IN THE DECODABLE REGION FOR A FINITE CODE

In finite-size quantum code, a critical concern is the engineering of code states to ensure their retention within the decodable phase, as depicted in Fig. 5 (b) of the main text. A viable strategy to achieve this involves conducting multiple rounds of stabilizer measurements during the state preparation phase. The underlying assumption in our prior analysis was the immediate encoding of logical information into the code state following a single round of

Stabilizer measurement \ Stochastic error	none	X error	X and Z error
Imperfect	$T_{\mathrm{pre}} = \omega(\log d)$	$T_{\mathrm{pre}} = \omega(\log d)$	$T_{\mathrm{pre}} \gg d$
Projetive	$T_{\mathrm{pre}} = O(1)$	$T_{\mathrm{pre}} = O(1)$	$T_{\mathrm{pre}} = O(1)$

TABLE S1. The overhead of $|\widetilde{++}\rangle$ state preparation under different circumstances. Notice that we consider only the rounds of measurements in state preparation T_{pre} here, to be distinguished with the rounds in an error correction cycle T. In principle projective measurement can prepare an arbitrary logical state if we chose the input state carefully, while imperfect measurement cannot directly preparation general states.

preparation, as described by Equation (S3). However, a more practical approach entails postponing the introduction of logical information until after several rounds of measurement and decoding. This method aims to enhance the resilience of the initial logical state, thereby increasing its robustness against potential errors.

In the specific context of quantum state preparation, our methodology initiates with the product $+$ states, targeting the establishment of the $|{+}{+}\rangle$ logical state. This process involves executing T_{pre} rounds of B_p POVM measurements. To simplify our model without loss of generality, we define the outcome of the first-round measurement as $\{s_p = +\}$, achieved by adjusting the sign of the stabilizers. During this procedure, stochastic Pauli X errors may occur. Subsequent to these measurements, a decoding step is conducted followed by the application of a recovery protocol, which yields an imperfect $|{+}{+}\rangle$ logical state. The resulting state, denoted as $|\widetilde{++}\rangle$, is characterized by an imperfection parameter approximately equal to $\beta_0 T_{\mathrm{pre}}$ on most plaquettes. This state is further subject to ancillary X logical errors, represented as X_l, and open-ended Pauli X string errors, denoted as X_c. Upon completion of these preparation steps, logical information is encoded by applying logical operators. After completing these code state preparation, the logical information is safeguarded through further error correction cycles, with performance evaluated using our theory outlined in earlier sections.

Unlike the perfect preparation case, the imperfect POVMs actually change the quantum state, i.e. they change the weight of the superposition of the opposite syndromes. Luckily, the final Pauli X recovery flips most of the unwanted superposition where $B_p = -1$ has a higher weight than $B_p = +$. The remaining unflipped syndromes are caused by open-ended X_c string near the T_{pre} time boundary, which are of the order $O(p^{|c|}e^{-4\beta_0})$ when both the measurement and stochastic error rates are small, where $|c|$ is the length of the string. While our prior analysis indicated a constant probability of logical X error chains in this process, the specific state $|\widetilde{++}\rangle$ remains unaffected by the logical X operator. Consequently, this allows for the high-probability preparation of a stable $|\widetilde{++}\rangle$ state.

Provided that we prepared an initial state with imperfect parameter $\sim \beta_0 T_{\mathrm{pre}}$, the robustness of the logical information is enhanced. The effectiveness of subsequent error correction cycles in safeguarding logical information is still described by Fig. 5 (b) of the main text with $\mathcal{T}_l = 1/\beta_0$ rescaled. To stay in the decodable region even when increasing code distance d, T_{pre} has to scale larger than $\log d$, $T_{\mathrm{pre}} = \omega(\log d)$. However, one should keep in mind that the measurement preparation procedure only works for logical X eigenstates, while other code states should be obtained by logical operation afterward. If one tries to directly prepare other states, the constant residual logical X error will damage the process. We also mention that our discussion above applies to starting from product $|0\rangle$ state and preparing with A_v measurement. In that case, we need to initialize a logical Z eigenstate first.

The complexity of toric code preparation is significantly increased due to imperfect measurement, in contrast to the ideal scenario involving perfect projective measurement. The protocol of multi-round syndrome measurements, initially proposed by Dennis et al. [5] to address stochastic measurement errors, serves a distinct function in the context of imperfect state preparation. This arises from the fundamental differences in the underlying SM obtained: RPGM in the work of Dennis et al. [5] and the SM model in our study, also refer to Tab. I of the main text. In the former scenario, the strategy leverages redundant syndrome data to decode and correct measurement errors, with the RPGM exhibiting a finite error correction threshold that enables suppression of erroneous strings. Conversely, in our investigation, the SM model lacks a finite phase capable of error correction, rendering direct preparation of generic logical states via imperfect measurements unfeasible. Nevertheless, preparing specific states in a finite size code, notably the logical X basis states, remains achievable through multi-round measurement processes. These processes are crucial for reducing the effective temperature ($\mathcal{T}_0 = 1/\beta_0$) of the model's two-dimensional component. Specifically, in the realm of imperfect measurements compounded by stochastic X errors, a preparation time $T_{\mathrm{pre}} = \omega(\log d)$ is

necessary to lower $\mathcal{T}_0$, thereby attenuating the emergence of non-local timelike error strings in subsequent error correction cycles. Note that we should distinguish the difference between the preparation time T_{pre} and the error correction cycle duration T. In Ref. [5] $T \gg d$ is required for a perfect code state under stochastic measurement and Pauli errors while they have not discussed about state preparation. In our case T_{pre} takes at least $\omega(\log d)$, and we anticipate $T \gg d$ is still necessary for the subsequent error correction cycle to further mitigate spacelike non-contractible error strings effectively.

Beyond the imperfect stabilizer measurements for X errors, stochastic Pauli Z errors may also occur during the preparation of the $\widetilde{|++\rangle}$ state. Consequently, it becomes necessary to repeatedly measure A_v stabilizers, which may also be subject to coherent noise, and to decode in order to mitigate logical Z errors. Fortunately, the physical product state $|+\rangle^{\otimes n}$ serves as an eigenstate for A_v stabilizers, rendering it an optimal initial state for addressing both Pauli Z errors and A_v syndromes. By mapping the Z decoding to an RPGM, one can argue that the logical Z errors are suppressed at low error rates, so the $\widetilde{|++\rangle}$ state preparation still works, although in that case we have to set $T \gg d$, as we are effectively performing an error correction cycle on Z errors. The overhead of $\widetilde{|++\rangle}$ state preparation is summarized in Tab. S1. Also, the preparation of $\widetilde{|00\rangle}$ could be tackled similarly using the X-Z duality. We also underscore that the 3D protocol for preparing specific logical Pauli basis states may not be applicable to more realistic preparation circuits, as will be discussed in the subsequent section. In this context, the further investigation of code space preparation becomes critically important and urgent.

SVIII. IMPERFECT CODE STATE OF THE REALISTIC CIRCUIT MODEL

Here we discuss the initial code state assuming the realistic B_{p_0} measurement circuit in Fig. 1(b) of the main text with imperfect 2-qubit gates. On each plaquette p_0, the measurement operator is expressed as Eq. (24) of the main text, i.e.

$$\begin{aligned}
M_+ &= \frac{1}{2}\left(1 + C_4 B_{p_0}\right)\left(1 + C_1 \sum_{i\in p_0}\sigma_i^z + C_2 \sum_{i<j\in p_0}\sigma_i^z\sigma_j^z + C_3 B_{p_0}\sum_{i\in p_0}\sigma_i^z\right).\\
C_4 &= \mathrm{e}^{-\mathrm{i}2t}\cos^4\frac{t}{2} + \mathrm{e}^{-\mathrm{i}2t}\sin^4\frac{t}{2}\\
C_1 &= -\frac{\mathrm{i}\mathrm{e}^{\mathrm{i}2t}\sin\frac{t}{2}\cos^3\frac{t}{2} + \mathrm{i}\sin\frac{t}{2}\cos^7\frac{t}{2} + \mathrm{i}\sin^7\frac{t}{2}\cos\frac{t}{2}}{\left(\mathrm{e}^{\mathrm{i}2t} + \cos^4\frac{t}{2}\right)^2 - \sin^8\frac{t}{2}}\\
C_2 &= -\frac{\sin^2\frac{t}{2}\cos^2\frac{t}{2}}{\mathrm{e}^{\mathrm{i}2t} + \cos^4\frac{t}{2} + \sin^4\frac{t}{2}}\\
C_3 &= \frac{\mathrm{i}\mathrm{e}^{\mathrm{i}2t}\sin^3\frac{t}{2}\cos\frac{t}{2} + \mathrm{i}\sin^3\frac{t}{2}\cos^5\frac{t}{2} + \mathrm{i}\sin^5\frac{t}{2}\cos^3\frac{t}{2}}{\left(\mathrm{e}^{\mathrm{i}2t} + \cos^4\frac{t}{2}\right)^2 - \sin^8\frac{t}{2}}
\end{aligned}\tag{S68}$$

Here we assumed all measurement results are $+$. It models the presence of coherent calibration error of two-qubit gates in a superconducting platform [?]. The imperfect initial state prepared by the above model is given by

$$\begin{aligned}
|\tilde{++}\rangle &\propto \left(\prod_{p_0} M_+\right)\bigotimes_{e_0}|+\rangle\\
&\propto \prod_{p_0}\left(1 + C_1\sum_{i\in p_0}\sigma_i^z + C_2\sum_{i<j\in p_0}\sigma_i^z\sigma_j^z + C_3 B_{p_0}\sum_{i\in p_0}\sigma_i^z\right)\\
&\times\left[\left(1 + C_4 {\prod}'_{p_0} B_{p_0}\right){\bigotimes}'_{p_0}\sum_{b_{p_0}=\pm}\left(1 + C_4 b_{p_0}\right)|B_{p_0} = b_{p_0}\rangle\right]\left[{\bigotimes}'_{v_0}|A_{v_0} = +\rangle\right]\bigotimes|++\rangle,
\end{aligned}\tag{S69}$$

The third line shows that the B_{p_0} syndromes are superposed as in Eq. (S17), which was the key point that leads to the failure of decoding in the simplified model. The second line can be viewed as additional coherent Z errors acting on the already imperfect state. They mainly affect the state's A_{v_0} components. Expanding the product in the second line, the result is a summation of Pauli Z chains with different coefficients. For simplicity, we examine the effect of a single Pauli chain $\prod_{i\in c}\sigma_i^z$. Each Pauli chain c can be decomposed into $\overline{Z}_l\prod_{p_0\in A}B_{p_0}\prod_{i\in l}\prod_{i\in c_{min}}\sigma_i^z$ where c_{min}

is the shortest chain that connecting the end points ∂c, $\overline{Z}_l = I, Z_{l_1}, Z_{l_2}$, or $Z_{l_1}Z_{l_2}$ could be the identity or logical Z operator depending on the logical class of c, and A is a set of plaquettes. The $\prod_{i\in c_{min}}\sigma_i^z$ part flip the sign of A_{v_0} at the endpoints, $|A_{v_0}=+\rangle \to |A_{v_0}=-\rangle$, while B_{p_0} operator only changes the phase of B_{p_0} basis $|B_{p_0}=+\rangle \to |B_{p_0}=+\rangle$,$|B_{p_0}=-\rangle \to -|B_{p_0}=-\rangle$ and leaves the probability of defect unchanged.

$$\begin{aligned}
&\prod_{i\in c}\sigma_i^z\left[\left(1+C_4\prod\nolimits'_{p_0}B_{p_0}\right)\bigotimes\nolimits'_{p_0}\sum_{b_{p_0}=\pm}(1+C_4 b_{p_0})\,|B_{p_0}=b_{p_0}\rangle\right]\left[\bigotimes\nolimits'_{v_0}|A_{v_0}=+\rangle\right]\bigotimes|{+}{+}\rangle \\
&\left[\left(1+C_4\prod\nolimits'_{p_0}B_{p_0}\right)\bigotimes\nolimits'_{p_0\notin A}\sum_{b_{p_0}=\pm}(1+C_4 b_{p_0})\,|B_{p_0}=b_{p_0}\rangle\bigotimes\nolimits'_{p_0\in A}\sum_{b_{p_0}=\pm}b_{p_0}\,(1+C_4 b_{p_0})\,|B_{p_0}=b_{p_0}\rangle\right] \\
&\left[\bigotimes\nolimits'_{v_0\notin\partial c}|A_{v_0}=+\rangle\bigotimes\nolimits'_{v_0\in\partial c}|A_{v_0}=-\rangle\right]\bigotimes\overline{Z}_l\,|{+}{+}\rangle
\end{aligned} \tag{S70}$$

Summing over all Pauli Z chains c, the main difference from Eq. (S17) is that A_{v_0} syndromes also becomes imperfect superposition instead of being exactly + (and additionally small coherent logical errors). Starting with such an imperfect code state leads to a worse QEC performance.

[1] G.-Y. Zhu, N. Tantivasadakarn, A. Vishwanath, S. Trebst, and R. Verresen, Nishimori's cat: stable long-range entanglement from finite-depth unitaries and weak measurements (2022), arXiv:2208.11136 [quant-ph].

[2] J. Y. Lee, W. Ji, Z. Bi, and M. P. A. Fisher, Decoding measurement-prepared quantum phases and transitions: from ising model to gauge theory, and beyond (2022), arXiv:2208.11699 [cond-mat.str-el].

[3] J. B. Kogut, An introduction to lattice gauge theory and spin systems, Rev. Mod. Phys. **51**, 659 (1979).

[4] S. Elitzur, Impossibility of spontaneously breaking local symmetries, Phys. Rev. D **12**, 3978 (1975).

[5] E. Dennis, A. Kitaev, A. Landahl, and J. Preskill, Topological quantum memory, Journal of Mathematical Physics **43**, 4452 (2002).

[6] H. Nishimori, Internal Energy, Specific Heat and Correlation Function of the Bond-Random Ising Model, Progress of Theoretical Physics **66**, 1169 (1981).

[7] C. Wang, J. Harrington, and J. Preskill, Confinement-higgs transition in a disordered gauge theory and the accuracy threshold for quantum memory, Annals of Physics **303**, 31 (2003).
